# Accelerometry Measuring the Outcome of Robot-Supported Upper Limb Training in Chronic Stroke: A Randomized Controlled Trial

**DOI:** 10.1371/journal.pone.0096414

**Published:** 2014-05-13

**Authors:** Ryanne J. M. Lemmens, Annick A. A. Timmermans, Yvonne J. M. Janssen-Potten, Sanne A. N. T. D. Pulles, Richard P. J. Geers, Wilbert G. M. Bakx, Rob J. E. M. Smeets, Henk A. M. Seelen

**Affiliations:** 1 Research School CAPHRI, Department of Rehabilitation Medicine, Maastricht University, Maastricht, the Netherlands; 2 Adelante, Centre of Expertise in Rehabilitation and Audiology, Hoensbroek, the Netherlands; 3 BIOMED Biomedical Research Institute, Hasselt University, Diepenbeek, Belgium; 4 Adelante Rehabilitation Centre, Hoensbroek, the Netherlands; 5 Department of Rehabilitation Medicine, Maastricht University Medical Centre, Maastricht, the Netherlands; University of Glasgow, United Kingdom

## Abstract

**Purpose:**

This study aims to assess the extent to which accelerometers can be used to determine the effect of robot-supported task-oriented arm-hand training, relative to task-oriented arm-hand training alone, on the actual amount of arm-hand use of chronic stroke patients in their home situation.

**Methods:**

This single-blind randomized controlled trial included 16 chronic stroke patients, randomly allocated using blocked randomization (n = 2) to receive task-oriented robot-supported arm-hand training or task-oriented (unsupported) arm-hand training. Training lasted 8 weeks, 4 times/week, 2×30 min/day using the (T-)TOAT ((Technology-supported)-Task-Oriented-Arm-Training) method. The actual amount of arm-hand use, was assessed at baseline, after 8 weeks training and 6 months after training cessation. Duration of use and intensity of use of the affected arm-hand during unimanual and bimanual activities were calculated.

**Results:**

Duration and intensity of use of the affected arm-hand did not change significantly during and after training, with or without robot-support (i.e. duration of use of unimanual use of the affected arm-hand: median difference of −0.17% in the robot-group and −0.08% in the control group between baseline and after training cessation; intensity of the affected arm-hand: median difference of 3.95% in the robot-group and 3.32% in the control group between baseline and after training cessation). No significant between-group differences were found.

**Conclusions:**

Accelerometer data did not show significant changes in actual amount of arm-hand use after task-oriented training, with or without robot-support. Next to the amount of use, discrimination between activities performed and information about quality of use of the affected arm-hand are essential to determine actual arm-hand performance.

**Trial Registration:**

Controlled-trials.com ISRCTN82787126

## Introduction

Stroke is a leading cause of serious, long-term disability. Four years after stroke, 67% of stroke patients perceive the non-use of their affected arm-hand as a major problem [1]. Loss of arm-hand function and, consequently, loss of arm-hand performance limits the execution of activities of daily living, leading to greater dependency, restricted social participation and decreased quality of life [2], [3]. Patients receive rehabilitation to improve arm-hand function, and even in the chronic stage after stroke, arm-hand performance may further improve with training [4], [5]. Robot-supported therapy is a rehabilitation method allowing patients to train their arm-hand with high intensity, a large amount of practice and minimal use of therapists' time. Several studies concluded that robot-supported arm-hand training may be a valuable rehabilitation method [6], [7], [8], [9], [10].

The ultimate goal of arm-hand rehabilitation is to improve the use of the affected arm-hand in the home situation of the patient during the execution of activities of daily living, i.e. improvement on ICF activity level [11]. Initially, many studies using robot-supported therapy focused on training and improvements at ICF function level [4], [7], [10]. However, literature has shown that a task-oriented training approach (ICF activity level) may be more effective in improving arm-hand skilled performance (AHSP) of stroke patients [12]. Some studies combined robot-supported arm-hand training with task-oriented training [13], [14], i.e. robot-supported training at ICF function level, followed by short periods of functional activity training (ICF activity level) without robotic support. Therapy in the control groups consisted of similar training at ICF function level without robot-support, followed by training of functional activities. In general, both groups improved on arm-hand performance as assessed by, among others, the FMMA (Fugl-Meyer Motor Assessment), MAL (Motor Activity Log) and ABILHAND [13], [14]. However, the robot-supported groups improved more than the control groups. Other studies investigated the effectiveness task-oriented robot-supported training (i.e. robot-assisted training on activity level). For example Houseman et al. investigated the effectiveness of task-oriented training with the robotic exoskeleton T-WREX in chronic stroke patients and found improvements in arm-hand use at ICF function level as well as ICF activity level [15]. In addition, several other studies showed improvements at ICF activity level [8], [13].

Next to the training method, the assessment of arm-hand performance is also very important when determining intervention effects. Multiple instruments are available to assess arm-hand performance at ICF activity level, i.e. tests to measure a person's capacity, perceived performance or actual performance [16]. Large differences exist between measurements in a standardized lab situation (capacity), perceived performance and actual performance in daily life [17], [18]. Although the ultimate goal of rehabilitation is to improve performance in the home situation, hardly any studies assess actual arm-hand performance, - i.e. improvements in arm-hand use in the home situation -, after robot-supported training. Only the study of Liao et al. [14] combined arm accelerometry, measuring arm activity ratio, with robotic training. Arm accelerometry can be used to determine actual performance and has been proven to be valid and reliable in measuring upper extremity activity in stroke patients [18], [19], [20]. Accelerometers have the advantage of being able to measure unobtrusively and continuously in daily life. Both duration of arm use and intensity of arm activity can be determined with accelerometers. Duration of arm use is defined as the time the arm and hand is active, expressed in minutes or expressed relative to the total time someone is awake.

Intensity of use is defined as the magnitude of arm activity, i.e. the total amount of activity expressed in counts [21].

The aim of the present study was to assess the extent to which accelerometers can be used to determine the effect of robot-supported task-oriented arm-hand training - relative to task-oriented arm-hand training alone - on the actual amount of arm-hand use of chronic stroke patients in their home situation

## Methods and Materials

### Participants and study protocol

**Figure 1:**
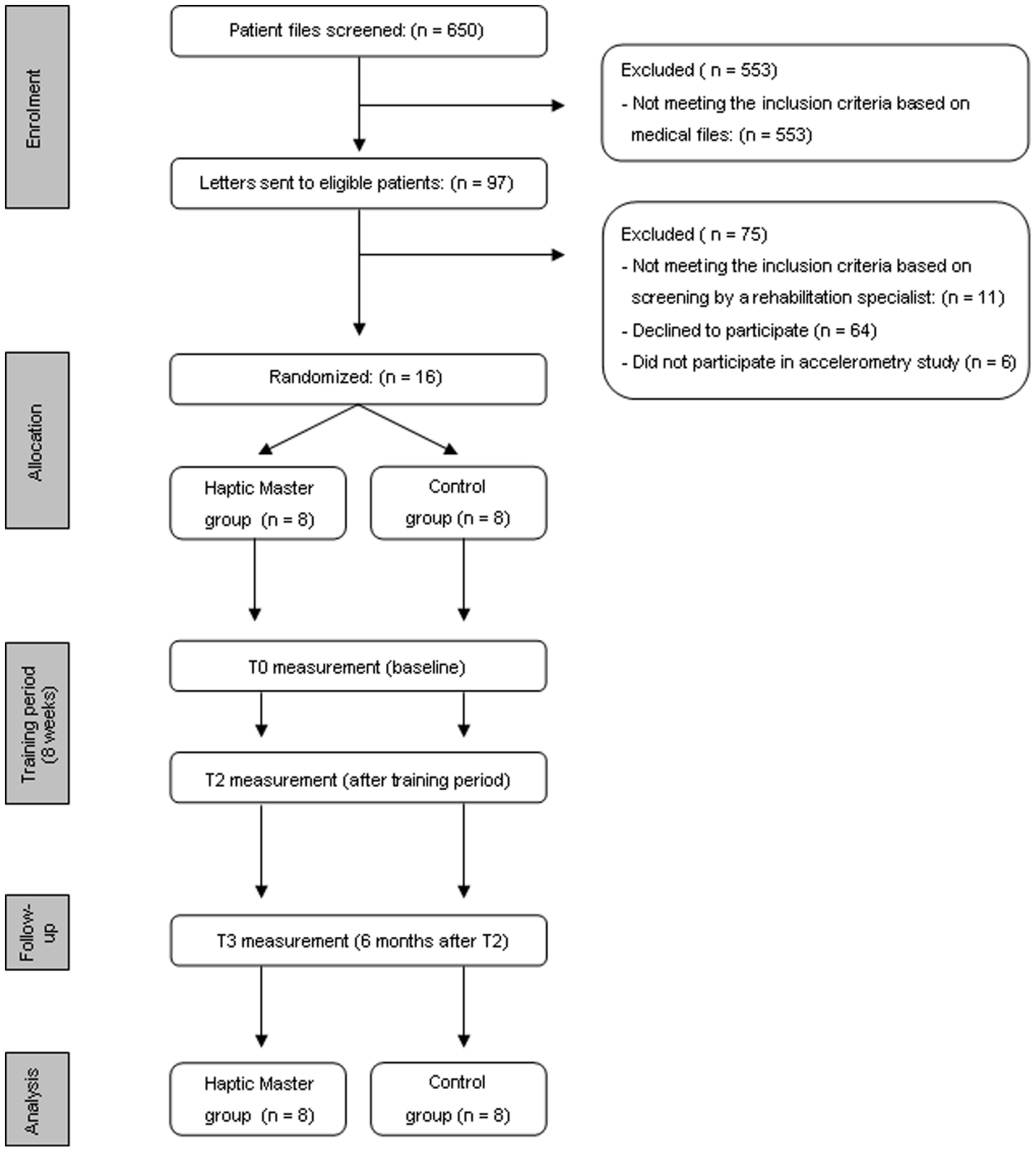
CONSORT flowchart. Flowchart representing the number of patients throughout the trial.

In the current study, which is a sub-study of a larger single-blind randomized controlled trial called TEST-TRACS (Technology-Supported Task-oriented Training of Arm-hand function in persons with Chronic Stroke, ISRCTN 82787126) [22], 16 chronic stroke patients, who all received their rehabilitation treatment at Adelante Rehabilitation Centre (Hoensbroek, The Netherlands), agreed to participate. Figure 1 shows the CONSORT flowchart representing the number of patients throughout the trial. The sample size of TEST-TRACS was determined after a power calculation based on results of a sensor-based intervention that used the same training method as the one in this study [23]. Assuming a clinical relevant improvement of 10%, an estimated standard deviation of 8% and, a two-sided alpha of 0.05 and a power of 0.80, twenty-two subjects should be included in the study. Sixteen of these twenty-two patients included in TEST-TRACS performed the measurements with the accelerometers and were therefore included in this sub-study. The inclusion criteria were: 1) first ever stroke, 2) 18–85 years old 3) clinically diagnosed with a central paresis of the arm-hand (strength: MRC grade 2–4 at entry into study), 4) post-stroke time ≥12 months, 5) normal cognitive level (Mini Mental State Examination (MMSE) score ≥26 [24]), 6) able to read and understand the Dutch language, 7) unable to fully perform, but motivated to train on, at least two of the following skills: ‘drinking from a cup’, ‘eating with knife and fork’, ‘taking money from a purse’ and ‘using a tray’. At the end of the inclusion period (i.e. in the last 6 months), inclusion criterion 4 was adjusted to post-stroke time ≥8 months, to improve patient inflow. The exclusion criteria were: 1) severe neglect (Bell Test [25], Letter Cancellation Test: minimum omission score of 15% [26]), 2) hemianopsia, 3) severe spasticity (Modified Ashworth Scale total arm >3), 4) severe additional neurological, orthopaedic or rheumatoid impairments which could interfere with task performance, 5) Broca aphasia, Wernicke aphasia, global aphasia (determined by the Akense Afasie Test [27], 6) apraxia (Apraxiatest of van Heugten [28]) and 7) attending another study/therapy to improve arm-hand function.

The participating rehabilitation physicians identified potential participants based on screening patients' medical files for inclusion and exclusion criteria. Letters containing information about the study and an invitation to participate were sent to potential participants. After stating their willingness to participate in the study, patients were screened by the rehabilitation physician for inclusion and exclusion criteria. After obtaining their informed consent, participants were included and were randomly allocated to either the group with robot-supported training (Robotic Rehabilitation (RR) group) or the control group, using blocked randomization (block size = 2). The randomization procedure was performed by an independent researcher using 2 opaque envelopes each containing a training condition code. Persons involved in data collection were blinded for group allocation. During the study period, participants were asked not to participate in other studies involving arm-hand performance. Training and data collection took place at Adelante Rehabilitation Centre (Hoensbroek, The Netherlands). All participants signed an informed consent prior to participating in the study. The criteria of the Helsinki declaration [29] were fulfilled.

### Ethics Statement

All (TEST-TRACS) study procedures were approved by the Medical Ethics Committee of Adelante. The TEST-TRACS trial is registered at www.controlled-trials.com (Unique identifier: ISRCTN82787126). The protocol for this trial and supporting CONSORT checklist are available as supporting information; see Checklist S1 and Protocol S1.

### Task-oriented training method

For training of the RR-group, the T-TOAT (Technology supported Task-Oriented Arm training) was applied, and for training in the control group the TOAT method (Task-Oriented Arm training) was applied [30]. The only difference between these methods is the use of the robotic system Haptic Master in T-TOAT. The (T-)TOAT method is based on part practice, i.e. the trained skills were divided into parts, which were first practiced in isolation and gradually combined into the complete task (chaining method [31]). The difficulty of the task parts was gradually increased and shaping principles were applied [32]. Variability of practice was, next to within-task exercise variety, also achieved by using multiple real life materials differing in size, structure, texture and fabric. The use of objects and the high number of repetitions facilitated coordination in task-related movements of the arm and hand [33], [34]. Specificity of practice was implemented by including tasks of daily living in a realistic environmental context to provide correct sensory information that elicited relevant problem solving strategies. The tasks were repeated frequently to accomplish a high intensity and a large amount of practice. Random practice (i.e. performing multiple tasks from different skills, in a random order) was implemented to enhance retention [35]. In addition, distributed practice was applied to avoid fatigue, optimize cognitive effort and support memory consolidation [36]. The motivation of the participants was increased by including real-world tasks which were meaningful and engaging to the participants [30]. Furthermore, an active role of the participant in the rehabilitation process was obtained through personal goal setting and control in the training program (exercise and material choice) to encourage motivation and treatment compliance.

### Robotic system

**Figure 2:**
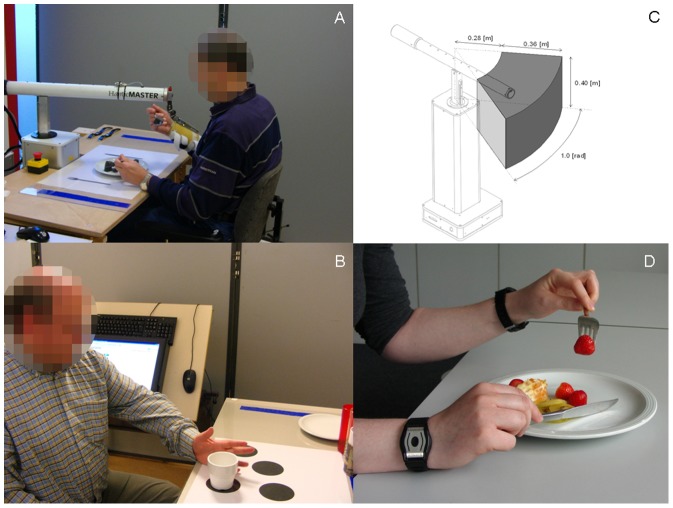
Set-up of the training, robotic device Haptic Master and Actiwatch. a) patient training in the robotic rehabilitation group, b) patient training in the control group, c) workspace of the Haptic Master (reprinted with permission from Van der Linde et al.[37]), d) patient wearing actiwatches.

The robotic system Haptic Master (MOOG, Nieuw-Vennep, NL) (figure 2) was used for the arm-hand training in the RR-group. Haptic Master is an end-effector based robotic device with three active degrees of freedom (DoF). A gimbal was attached to the Haptic Master connecting the forearm of the patient with the Haptic Master, thereby adding 3 extra (non-actuated) degrees of freedom. This gimbal was specifically designed for the task-oriented training, allowing the hand to be free to grasp and manipulate real objects in the three dimensional space. The Haptic Master is suitable for medium sized workspaces [37] and tasks in both sitting and standing position can be performed. Training with Haptic Master was possible in both passive mode and active mode. A software program called the Haptic-TOAT, was developed at Adelante (in cooperation with Zuyd University, Heerlen, The Netherlands) to implement the task-oriented training method T-TOAT [30]. Details on the use of Haptic Master have been reported by Timmermans et al [30].

### Arm-hand training program

For both the RR-group and the control group, training was provided during 8 weeks, 4 times/week, 30 min twice daily (separated by 0.5–1 hour of rest). At baseline, participants chose a minimum of 2 out of 4 skills to train: ‘drinking from a cup’, ‘eating with knife and fork’, ‘taking money from a purse’ or ‘using a tray’. Before training, participants were educated about the principles of task-oriented arm-hand training and the importance of frequent training to enlarge therapy success. Video-instructions were used to explain the exercises. The training program was similar for both groups, the only difference being the use of the robotic Haptic Master device in the RR-group. Training was provided by a physiotherapist, occupational therapist or movement scientist.

### Outcome measures

The demographic data obtained from the medical files was: age, gender, date and type of stroke, side of hemiparesis, and hand dominance. Outcome measurements were taken upon entry into the study (T0, baseline), after 4 weeks training (T1) at the end of the 8 weeks training program (T2) and 6 months after finishing the training program (T3) [22]. For the present paper, arm accelerometry data obtained at T0, T2 and T3 was used as a measure to assess actual arm-hand use. The baseline measurements of the FMMA, Action Research Arm Test (ARAT) and MAL were used to describe arm-hand function at the start of the study. Persons who performed the assessments were blinded for treatment allocation and where neither involved in the training nor the data analysis. A diary was kept during training sessions by the person who provided the training, to determine therapy compliance. Compliance was determined as the percentage of training sessions patients attended relative to the total of 64 training sessions.

### Accelerometer data processing and analysis

Arm accelerometry can be used to determine actual performance and has been proven to be valid and reliable in measuring upper extremity activity in stroke patients [18], [19], [20]. In this study, participants wore Actiwatch-AW7 devices, containing a uniaxial piezoelectric accelerometer (CamNtech Ltd, Cambridge, UK), on both wrists. Sample frequency was 32 Hz and accelerations over 0.05 g were recorded. Analogue to digital conversion was 8 bits. From the accelerometer signal, maximal signal intensity per second (I_max/sec_ i.e. the highest peak within each consecutive second) was identified. Next, for every 2 consecutive I_max/sec_, signal amplitudes of these two peaks were summed. The resultant is expressed in units of ‘counts’ [21]. In essence, the ‘raw’ accelerometer signal was transformed into an ‘activity count signal’. The data was imported using the software Sleep-&-Activity-Analysis 11.7 (CamNtech Ltd, Cambridge, UK) and were further analysed using Matlab version 2006a (The MathWorks Inc., Natick, MA).

**Figure 3:**
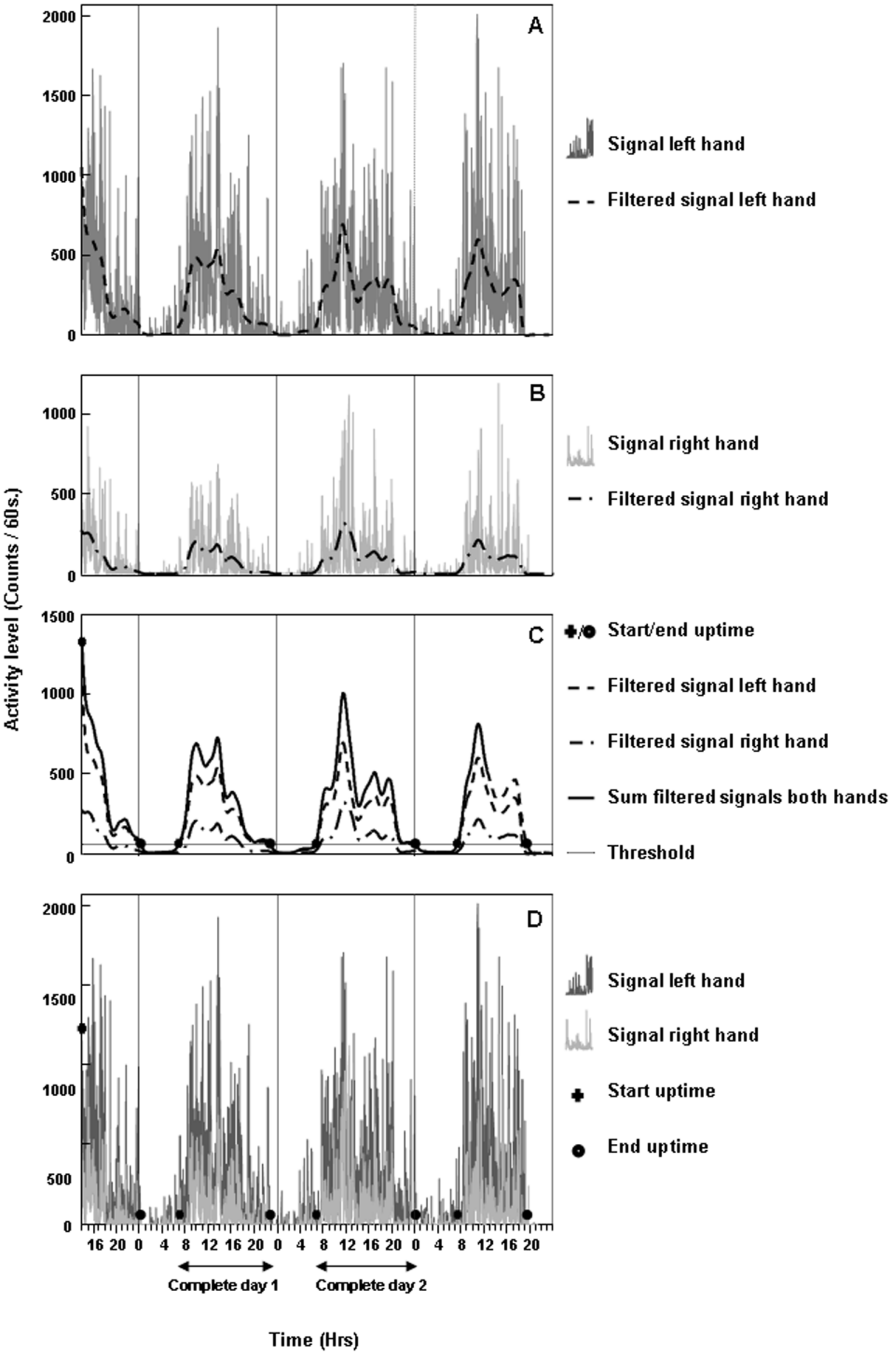
Data processing. A) Raw activity count data (solid) and filtered data (dashed) of the left hand; B) Raw activity count data (solid) and filtered data (dashed) of the right hand; C) Filtered data of both hands and summation of filtered data, including start points (+) and endpoints (O) of uptime; D) Raw activity count data of both hands including start points (+) and endpoints (O) of uptime.

Patients wore the wrist accelerometers for 72 consecutive hours (an incomplete day, followed by two complete days and an incomplete day). During the arm-hand training, the accelerometers were taken off, as arm-hand use during training did not reflect the actual arm-hand use at home. Only data from the two complete days were used for analysis. As accelerometers were waterproof, there was no need to take them off during, for instance, bathing. Uptime (T_up_) was defined as the time span between first arm activity detected in the morning and last arm activity detected in the evening/night. To determine the uptime, the activity count signals of both arms were filtered (figure 3a and b) using a 4^th^ order zero time-lag low-pass Butterworth filter. Cut-off frequency was 0.0025 Hz. However, in a few cases where signal variability was still too high, a cut-off frequency 0.00125 Hz was applied to correctly identify the uptime. Next, the filtered signals of the 2 arms were summed (figure 3c). Using the resultant signal, first and last arm activity during each day was detected by setting the minimal signal amplitude ( = threshold), indicating genuine arm activity, at 10 counts/min. This threshold was computed, based on signal variance recorded during rest periods. The first data point crossing this threshold, which was followed by prolonged activity levels well above the threshold (thus disregarding short epochs of e.g. rest), was identified as the start of the uptime. Similarly, end of uptime was identified as the last data point before the signal crossed the threshold, followed by a prolonged period of sub-threshold signal intensity. Night time data was discarded. The raw activity signals of both arms (figure 3d) were used for all further calculations.

Actual amount of arm-hand use was calculated in several ways, focusing on the duration of use and the intensity of use of the affected arm-hand. In order to be able to investigate the use of the affected arm-hand alone, or in combination with the unaffected arm-hand, a distinction was made between unimanual use and bimanual use. Duration of use is expressed as ‘hours of arm-hand use relative to the uptime’, since skills are generally performed during waking hours. Duration of use of the affected arm-hand was subdivided into a) duration of use during unimanual activity of the affected arm-hand and b) duration of use during bimanual arm-hand activity. Intensity of use was defined as the sum of counts (signal intensity per data point) within a given time epoch, thus producing the ‘area under the curve’. For both the affected arm-hand and the unaffected arm-hand, intensity of use was calculated as total intensity. For the affected arm-hand, intensity of use was further subdivided into a) intensity of use during unimanual activity of the affected arm-hand and b) intensity of use during bimanual arm-hand activity. In addition, the ratio between total intensity of the affected arm-hand and total intensity of the unaffected arm-hand was calculated. Table 1 gives an overview of the variables regarding actual amount of arm-hand use and how they were calculated.

**Table 1:** Variables used to calculate the actual amount of arm-hand use.

Abbreviation (unit)	Definition
T_up_ (hour)	Time span between first arm activity detected in the morning and last arm activity detected in the evening/night, displayed in hours
D_aff-uni_ (%)	(Hours with only activity of the affected arm-hand)/(T_up_) * 100%
D_bi_ (%)	(Hours with bimanual arm-hand use)/(T_up_) * 100%
I_tot-aff_ (activity/hour)	(Sum of acceleration counts of the affected arm-hand during unimanual activity of the affected arm-hand and during bimanual activity)/(hours with only activity of the affected arm-hand + hours with only bimanual activity)
I_tot-unaff_ (activity/hour)	(Sum of acceleration counts of the unaffected arm-hand during unimanual activity of the unaffected arm-hand and during bimanual activity)/(hours with only activity of the unaffected arm-hand + hours with only bimanual activity)
I_aff-uni_ (activity/hour)	(Sum of acceleration counts of the affected arm-hand during unimanual activity of the affected arm-hand)/(hours with only activity of the affected arm-hand)
I_aff-bi_ (activity/hour)	(Sum of acceleration counts of the affected arm-hand during bimanual activity)/(hours with only bimanual activity)
R_tot-aff/tot-unaff_	Ratio of I_tot aff_ divided by I_tot unaff_

### Statistical data analysis

Because data was not normally distributed, the data was analysed using non-parametric tests with SPSS software (SPSS Inc., Chicago, IL). Baseline differences between groups regarding nominal data (gender and dominant hand impaired) were tested with a Fisher's Exact Test. Other baseline differences between groups were determined with the Mann-Whitney U-test. Within-group differences were analysed using Friedman tests and Wilcoxon signed rank tests. Alpha was set at 0.05. For multiple comparisons (T0–T2 and T2–T3), Bonferroni correction was applied, resulting in an alpha value of 0.025. For between-group differences, difference scores between two measurements were calculated, i.e. T2 minus T0; T3 minus T2. These difference-scores reflect within-group changes regarding progress in arm-hand use, and were tested between groups using Mann-Whitney U-tests. Intention-to-treat analysis was applied.

## Results

Sixteen chronic stroke patients were included between May 2009 and May 2011. A flowchart is shown in figure 1.

### Patient characteristics

Patient characteristics and baseline levels are shown in table 2. Five patients were included with a post-stroke time shorter than the initially required 12 months (i.e. RR-group: 10 months (N = 1) and 8 months (N = 1) post-stroke, control group: 11 months (N = 2), 10 months (N = 1) post-stroke). No patients dropped-out. All 16 participants were included in all analyses. One patient (in the RR-group) fainted briefly once. However, after visiting the medical specialist, the cause turned out to be a change of medication. No relationship with the intervention was found. No adverse effects of the study were found.

**Table 2:** Overview of patient characteristics at baseline.

		Robotic Rehabilitation group (n = 8) median [IQR]	Control group (n = 8) median [IQR]	p-value
**Age (years)**		63.5 [55.0, 68.0]	55.0 [50.5, 60.5]	p = 0.195
**Gender**	**Male (n)**	5	5	p = 1.000
	**Female (n)**	3	3	p = 1.000
**Post stroke time (months)**		12.5 [11.0, 16.0]	25.5 [12.5, 56.5]	p = 0.105
**Dominant hand impaired**		5	2	p = 0.315
**Fugl-Meyer Motor Assessment**		49.5 [36.0, 55.5]	52.5 [45.5, 58.0]	p = 0.442
**Action Research Arm Test**		31.5 [26.5, 38.5]	40.0 [25.5, 48.0]	p = 0.442
**Motor Activity Log**		3.57 [2.82, 4.73]	4.55 [3.00, 6.54]	p = 0.574
**Mini Mental State Examination**		29.0 [27.0, 30.0]	29.5 [29.0, 30.0]	p = 0.382
**D_aff-uni_**		0.91 [0.39, 2.07]	1.01 [0.51, 3.07]	p = 0.505
**D_bi_**		49.38 [45.05, 54.52]	68.27 [54.54, 69.90]	p = 0.050
**I_tot-unaff_**		17826 [15329, 18947]	17728 [15374, 20986]	p = 0.645
**I_tot-aff_**		7634 [6261, 8466]	10186 [9043, 11131]	p = 0.005

IQR  =  Inter Quartile Range; D_aff-uni_  =  duration of unimanual use of the affected arm hand; D_bi_  =  duration of bimanual use; I_tot-unaff_  =  intensity of use of the unaffected arm-hand; I_tot-aff_  =  intensity of use of the affected arm-hand; NS  =  not significant.

At baseline, patients in the RR-group and the control group did not differ significantly as to the demographic variables. Arm-hand function, as determined with the FMMA, ARAT and MAL did not differ significantly between groups. The duration of use of the affected arm-hand during unimanual activity (D_aff-uni_) and the intensity of use of the unaffected arm-hand (I_tot-unaff_) did not differ significantly between the RR-group and the control group at baseline. However, the duration of use of the affected arm-hand during bimanual activity (D_bi_) and the intensity of use of the affected arm-hand (I_tot-aff_) were significantly lower in the RR-group compared to the control group. Compliance of the patients to attend the training sessions was comparable between the RR-group (96.0% of the maximal number of sessions) and the control group (96.2% of the maximal number of sessions).

### Duration of arm-hand use

**Figure 4:**
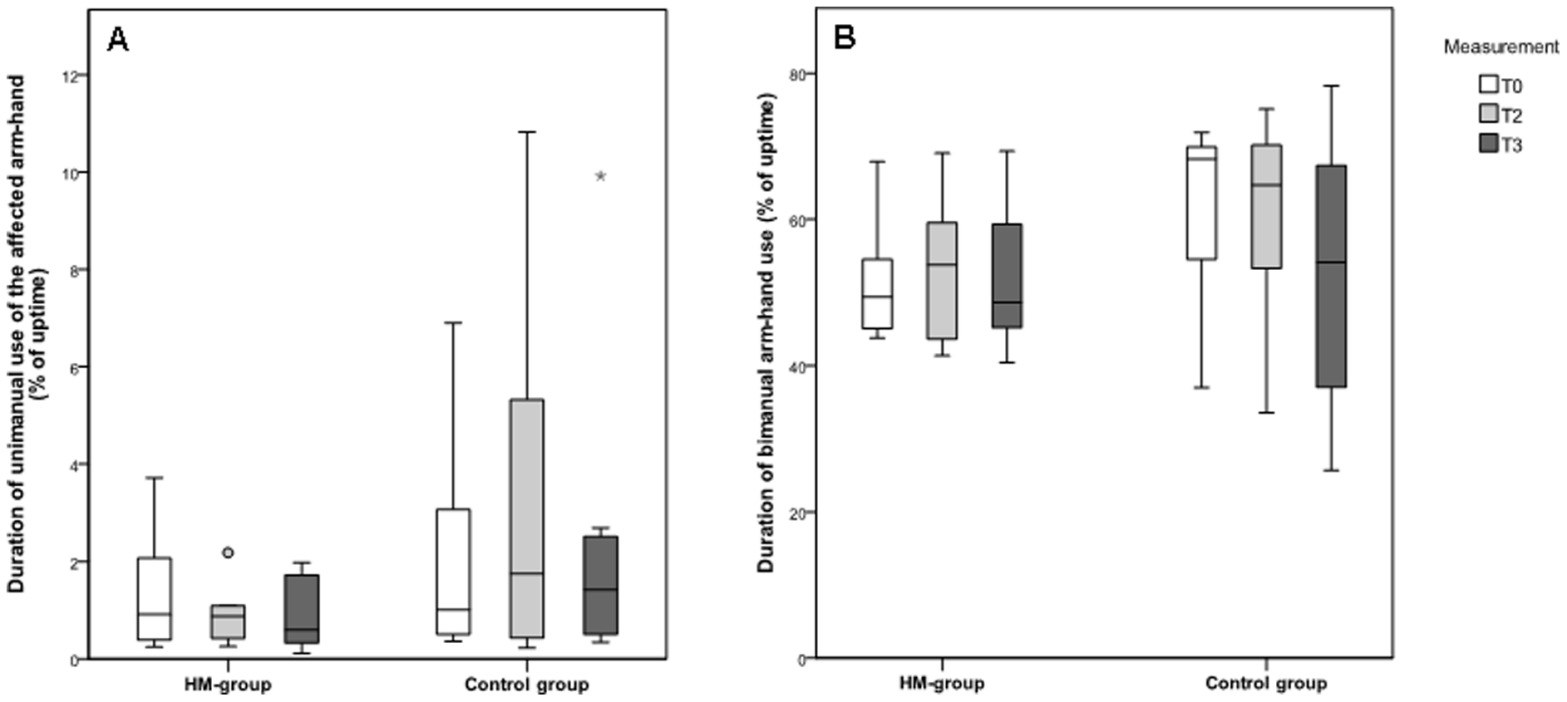
Duration of arm-hand use. Boxplots of the duration of a) unimanual use of the affected arm-hand and b) bimanual use, expressed as a percentage of uptime. The circle represents an outlier and the star represents a far out value.

The duration of unimanual use of the affected arm-hand (D_aff-uni_) and the duration of bimanual use (D_bi_), expressed as a percentage of uptime, at three time points, are presented in figure 4. For the RR-group, the median duration of unimanual use of the affected arm-hand (D_aff-uni_) was 0.91% at T0, 0.88% at T2 and 0.59% at T3, and for the control group 1.01% at T0, 1.75% at T2 and 1.42% at T3. For the RR-group, median duration of bimanual arm-hand use (D_bi_) was 49.38% at T0, 53.74% at T2 and 48.63% at T3, and for the control group 68.27% at T0, 64.70% at T2 and 54.14% at T3.

No significant between-group differences were found for both D_aff-uni_ and D_bi_ between end of the training and baseline (T2–T0; p = 0.57 for D_aff-uni_ and p = 3.82 for D_bi_) or between follow-up and the end of the training (T3–T2; p = 1.00 for D_aff-uni_ and p = 0.88 for D_bi_).

Both the RR-group and the control group showed no significant within-group changes in duration of unimanual use of the affected arm-hand and duration of bimanual arm-hand use between the start of the study (T0) and after 8 weeks training (T2) (p≥0.311). Also, between the end of the training (T2) and follow up (T3), no significant within-group changes were found for the RR-group and for the control group (p≥0.148).

Patients in both groups used their affected arm-hand significantly longer during bimanual activity compared to unimanual activity, at all measurement moments (p<0.05).

### Intensity of arm-hand use

**Figure 5:**
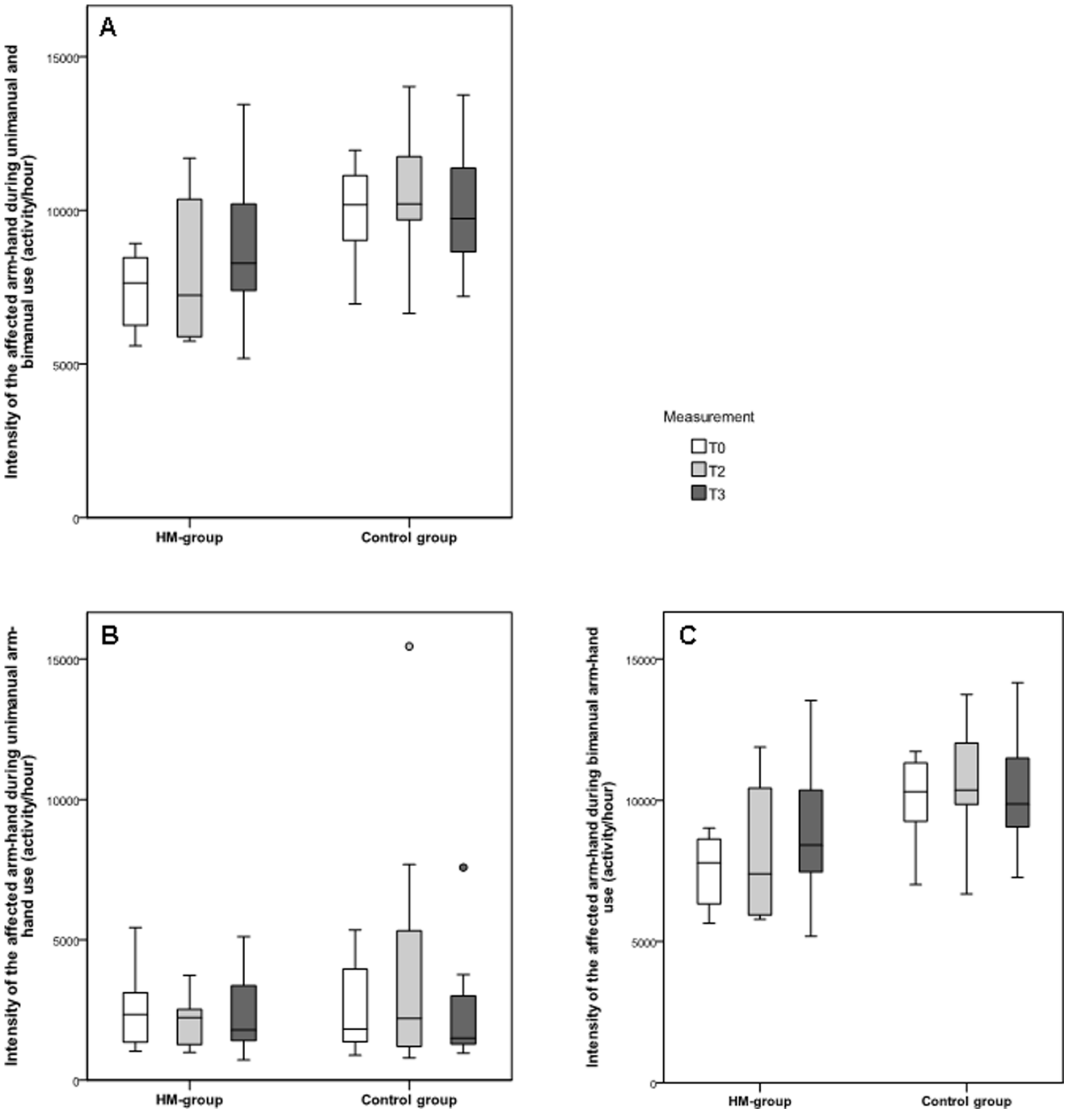
Intensity of use of the affected arm-hand. Box plots of the intensity of use of the affected arm-hand during a) both unimanual and bimanual activity, b) unimanual activity of the affected arm-hand and c) bimanual activity. Circles represent outliers.

The intensity of use of the affected arm-hand (I_tot-aff_) is displayed in figure 5a. No significant between-group differences were found for I_tot-aff_ between baseline and the end of the training (T2–T0; p = 0.88) or between follow-up and the end of the training (T3–T2; p = 0.20). For both the RR-group and the control group, no significant within-group changes were found for the intensity of use of the affected arm-hand (I_tot-aff_) between baseline, after 8 weeks training and at follow-up (p≥0.313).

The differentiation in intensity of use during unimanual activity of the affected arm-hand (I_aff uni_) and the intensity of use during bimanual arm-hand activity (I_aff bi_) is shown in figures 5b and 5c.

Figure 5b shows the intensity of the affected arm-hand for unimanual use, while figure 5c shows the intensity of the affected arm-hand for bimanual use. For the RR-group, the median intensity of unimanual use of the affected arm-hand (I_aff-uni_) was 2334 counts at T0, 2229 counts at T2 and 1785 counts at T3, and for the control group 1824 counts at T0, 2201 counts at T2 and 1489 counts at T3. For the RR-group, the median intensity of the affected arm-hand during bimanual arm-hand use (I_aff-bi_) was 7781 counts at T0, 7395 counts at T2 and 8410 counts at T3, and for the control group 10297 counts at T0, 10360 counts at T2 and 9863 counts at T3.

No significant between-group differences were found for either I_aff-uni_ (fig 5b) or I_aff-bi_ (fig 5c) between baseline and the end of the training (T2–T0; p = 0.80 for I_aff-uni_ and p = 1.00 for I_aff-bi_) and between the end of the training and follow-up (T3–T2; p = 0.65 for I_aff-uni_ and p = 0.23 for I_aff-bi_). In addition, no significant within-group differences were found for the intensity of the affected arm during unimanual use (I_aff-uni_) or during bimanual use (I_aff-bi_), between all measurement moments for both the RR-group and the control group (p≥0.208).

For both groups, the intensity of use of the affected arm-hand was significantly higher during bimanual tasks compared to unimanual tasks, at all time points (p<0.05).

The ratio between the intensity of the affected arm-hand and the intensity of the unaffected arm-hand did not exceed 1.0 in any of the patients nor any of the measurements (data not shown). Furthermore, no significant between-group differences were found regarding the ratio (p≥0.674). Also, the ratio did not change significantly over time in either group (p≥0.669).

## Discussion

The aim of the present study was to assess the extent to which accelerometers can be used to determine the effect of robot-supported task-oriented arm-hand training relative to task-oriented arm-hand training alone, on the actual amount of arm-hand use of chronic stroke patients in their home situation. Accelerometer data was used to determine the actual amount of use in terms of both duration of use and intensity of use.

In both the robot-supported group and the control group, no improvements were found with respect to duration of use and intensity of use of the affected arm-hand after 8 weeks intensive task-oriented training and at follow-up. This is in contrast with results from a clinical trial performed by Liao et al. who found an improved arm activity ratio after robot-supported training [14]. However, their patients had a more severely affected arm-hand function, probably resulting in a larger effect size regarding training [38]. Furthermore, their training was more intensive (though shorter) and featured another robotic system (Bi-Manu-Track). The overall time spent on therapeutic activity in Liao's study was similar to that in the present study. Furthermore, the patients of Liao et al. received robot-supported training at ICF function level, followed by 15 minutes training at ICF activity level without robot-support. In contrast, the RR-group patients in our study received only task-oriented robot-supported training, and no additional training without the robotic system. Moreover, there were some differences between the two studies in how actual arm-hand use was measured. For instance, Liao et al. used triaxial accelerometers compared to uniaxial accelerometers in the present study. In addition to the arm activity ratio calculated in both studies, the present study also investigated the activity of the affected arm and the unaffected arm separately, and distinguished between unimanual and bimanual use of the arm-hand.

The fact that the present study found no additional effects of the robot-supported training is in line with a study of Kahn et al, where similar effects were found for the robot-supported reaching group and the unassisted reaching group [39]. Patients included in the present study had a high level of arm-hand function. This might be a reason why no effects of the robot-supported training were found. Patients with a lower level of arm hand function might benefit more from the robot-supported training because the robotic support might reduce problems associated with e.g. muscle weakness [40]. However, an additional effect in the RR-group could be expected from the haptic feedback, as feedback is known to be an important component of motor learning [41].

In line with Michielsen et al [42], the present study revealed that the affected arm-hand was used more during bimanual tasks compared to unimanual tasks, for both duration and intensity of use.

Other results from the current randomized clinical trial, reported by Timmermans et al. [22], showed significant improvements on ICF activity level; i.e. significant improvements on the ARAT between baseline and the end of the training for the RR-group and significant improvements on the MAL for both groups. However, despite an improvement in arm-hand capacity (measured with the ARAT) and perceived performance (measured with the MAL), no improvements on actual arm-hand use as measured by accelerometry were found. Several explanations may be given regarding for why patients perceive themselves to be using their arm-hand more at home, in contrast to the data on their actual use of arm-hand, as measured objectively with accelerometers in the present study. First of all, patients' perception of performance is apparently not only guided by amount of use, but also by other, more quality-related reflections regarding their arm-hand performance. Amount of use provides information about the duration and intensity of arm-hand use. It must be considered that a greater amount of use may not necessarily reflect a better quality of performance. Secondly, the activity, registered during 2 entire days, will not only include task-related activities, but also non-functional movements, unintentional activity, and arm activity as a result of general body movement (e.g. during walking). Accelerometry as used in the present study is not capable of discriminating between activities or specific activity-related arm-hand movements and general or unintentional arm-hand movements. Discrimination between such activities would, however, be very interesting in order to detect what kind of activities the patients perform, and whether or not patients changed or improved their activities or started to perform new activities (i.e. activities they were not able to perform before the intervention) with their affected arm-hand as a result of the training. If a patient can perform an activity he could not perform before intervention, this is a positive effect of the training. But if this new activity is performed instead of another activity, the total activity count measured with accelerometers might not change, thus obscuring any positive effects. Fortune and co-workers combined accelerometry with gyroscopes and energy-expenditure to categorise activities, based on their activity level [43]. A combination of accelerometers, gyroscopes and magnetometers has been shown to be useful in recognising activities involving the lower limb, such as walking, jumping and cycling [44]. The use of multiple sensors may also be useful for recognising activities involving the arm-hand [45], [46].

Next to the amount of use, quality of use is also an important variable in assessing arm-hand performance. Quality of use may cover different constructs, for instance, the physical effort needed to perform a skill, the efficiency, safety or independence with which a skill is performed or parameters describing the execution of the movement such as velocity or smoothness [47].

### Study Limitations

Several methodological considerations related to the present study exist. Firstly, despite the randomisation procedure, participants in the RR-group demonstrated less arm-hand activity at baseline in terms of duration of use of the affected arm-hand during bimanual activity (D_bi_) and the intensity of use of the affected arm hand (I_tot-aff_). It is yet unknown to what extent this might have influenced our results. However, no significant between-group differences exist at baseline regarding demographics and arm-hand function as measured with clinical tests. Furthermore, for the between-group analysis the difference in I_tot-aff_ at baseline is taken into account by calculating difference scores between measurements.

Secondly, 5 patients were included with a post-stroke time <12 months. However, since all patients were in the chronic phase after stroke, i.e. >6 months post-stroke [48], [49], it is unlikely that this has influenced the results.

Thirdly, the low sample size combined with high variance could have caused the lack of statistical significance in improvements in actual amount of arm-hand use. The high variance is probably caused by the fact that the actual arm-hand use was measured during daily life and not in a standardized environment. Low sample size can be explained by the intensity of the training program (i.e. 4 times a week, for 8 weeks in total). This high intensity resulted in a rather small number of patients willing to participate.

Lastly, there was a between-group difference in the number of patients whose dominant hand was affected. It would have been plausible to expect patients whose dominant hand is affected, to use their affected arm-hand more than patients whose non-dominant hand is affected. However, at baseline the contrary was observed.

### Conclusion and future research

Accelerometers, as used in the present study, did not reveal significant within-group changes for either duration or intensity of actual arm-hand use following task-oriented arm-hand training, with or without robot-support. In addition to the amount of use, additional information to discriminate between specific activities and information about the quality of use of the affected arm-hand is essential to further elucidate (the quality of) actual arm-hand performance. Future research on actual arm-hand performance should assess these quality-related phenomena. However, the instruments to do so are still lacking [16]. Research should therefore focus on the use of other sensors, additional to accelerometers, and advanced data analyses methods, in order to provide information about the performance of specific activities and the quality of use.

## Supporting Information

Protocol S1 **Trial protocol.**
(PDF)

Checklist S1 **CONSORT checklist.**
(PDF)
