# Supplementary material for: Accelerometry Measuring the Outcome of Robot-Supported Upper Limb Training in Chronic Stroke: A Randomized Controlled Trial
Source: PLoS One. 2014 May 13;9(5):e96414. doi: 10.1371/journal.pone.0096414 (PMC4019639; doi:10.1371/journal.pone.0096414)
Supplement: Protocol S1 — Trial protocol. (PDF) [file pone.0096414.s001.pdf]

TEST-TRACS

Technology-Supported Task-oriented Training of Arm-hand function in persons with Chronic Stroke

Studie naar het effect van technologie-ondersteunde arm- en handvaardigheidstraining bij mensen met een CVA

27-10-2009

1. Introduction: Problem definition and project aims.

Upper extremity skills training in stroke

Stroke is the third leading cause of death in the world and an important cause of hospital admission, long-term disability and high health costs [1] [2]. Over one million people in USA and a quarter of a million in UK are living with long-term disability resulting from stroke [3] [4]. In the Netherlands every year 162/100.000 persons get a stroke, which leads to 25000 stroke incidents a year [5].
As age of the population increases with improving health care [6], an increase of stroke incidence from the mid-nineties until today has been observed. This tendency is likely to increase in the future [7].

Approximately 80% of acute stroke patients suffer from acute hemiparesis [8] and from cognitive function loss [9]. This unilateral motor deficit leads in approximately 40% of stroke patients to chronic upper extremity impairment, limiting functional use as well as engagement in community life [10] [8]. Although posture and gait tend to improve, recovery of arm-hand function (AHF) strongly lags behind recovery of other functions. Poor arm hand performance is associated with poor quality of life after stroke [11].

Persons after stroke can realize functional gains any time after stroke as latent sensorimotor functions can be realized with a pulse of goal-directed therapy [12].
In the Netherlands, traditional exercise concepts (e.g. Bobath, Perfetti) for stroke rehabilitation are followed by 80% of physiotherapists, even though there is evidence that e.g. motor relearning programs are better [13] [14] [15].
Rehabilitation methods after stroke have been evolving during the last 15 years from analytical rehabilitation methods to task-oriented training approaches. Focus of treatment has been shifted from spasticity reduction to strength training and improving motor control [12] [16], as is shown e.g. by development of new treatment approaches such as task-oriented training, mental practice and constrained-induced movement therapy (with its task-oriented "shaping" principles).

Task-oriented training is a repetitive training of real world activities that is advocated because of its positive effect on arm-hand function recovery (motor control and strength) in persons after stroke [17] [18] [19] [20] [21] [22] [23].

A large repetition of goal-directed, environmentally contextual and ever-changing training which includes anticipatory locomotor adjustments, goal oriented behavior & cognitive processing is used to obtain increase in muscle power and more efficient movement strategies for functional task performance [20] [16, 24] [25] [14]. Task oriented training is proven to have a faster and better treatment outcome than Bobath therapy [15]. An advantage of task-oriented training is that the patient can choose to train skills that are difficult in his personal every day life. This will increase intrinsic motivation [12], which in turn will be of great benefit for exercise compliance.
Stroke patients have not reached their full potential when they are discharged from hospital [25]. However, after discharge there seem to be little services available for persons after stroke, leading to high levels of patient dissatisfaction [26] [27]. Outpatient physiotherapy rehabilitation is typically only 2-3 times per week. Guided home rehabilitation leads to further improvement [28] and gives important advantages: the patient can train much more often which will lead to better results and faster progress in motor learning [29]; the patient can train in a comfortable home setting; and paramedical staff will be supported and relieved from work load which will reduce governmental costs for health services. Acceleration of recovery will provide higher quality of life for persons after stroke.

The ageing of the population with its increasing incidence of stroke victims, the shift in stroke treatment from an analytic to a task-oriented approach and the latent potential for further recovery in chronic stroke patients has led to the development of technology supported training and telerehabilitation to allow additional training in a home-setting and its remote guidance by skilled therapists. Multidisciplinary approaches (by e.g. physiotherapists, neurologists, engineers, computer programming experts) have been started to make new training possibilities, such as robotic techniques for rehabilitation, virtual reality training systems and telerehabilitation.
The development of new training techniques focuses on the one hand on robotic systems that are acting on upper limb movement by supporting or resisting forces. On the other hand there is the development of systems that monitor motor performance and store their information in base stations. The latter can process the information and feed it back to the therapist(s) and the patient. Monitoring systems can be run on a central server, so that information can be accessed through the web by patients and health-carers.
Robotic training, virtual reality training and web based processing and feedback can be used in combined applications.

Position of proposed RCT study in the PhD study

The study described in this proposal is the third sub-study of the PhD project of A. Timmermans.

General aims of the total PhD-project are:

- Development of an assessment/training protocol and training system for skilled arm hand function in persons after stroke;
- Assessment of the added value of the developed system for skilled arm hand function in a rehabilitation setting relative to therapy without technology support.

The *relevance of the total PhD-project* lies in its clinical contribution to the development of a technology supported task-oriented training system for arm- & hand function in stroke patients, which will be validated by clinical trial(s). Haptic Master should enable persons after a stroke to train independently, more frequently and for longer duration to improve functional outcome and provide faster functional progress in arm hand function. It should also enable the therapist to remotely evaluate treatment progress.

Relevance of the RCT study, proposed in this protocol:
Aim of this longitudinal clinical study (sub-study 3) is to evaluate the therapeutic value of technology-support for arm hand function treatment outcome and treatment compliance.
Previous sub-studies:

- In a first sub-study, 40 persons after stroke were interviewed to find out which are their skill training preferences. A journal paper to report this study has been published in Journal of Disability and Rehabilitation 2009, 31 (16): 1344-1352.
- A literature review on technology assisted rehabilitation of arm and hand function in stroke patients is in progress. A journal paper has been published in Journal of NeuroEngineering and Rehabilitation 2009, 6:1.
- Functional analyses of 10 arm-hand skills, selected from the chronic stroke patient gross-list from the first sub-study have been performed, and training protocols for these 10 skills have been developed.
- The training method that is behind the exercise protocols (T-TOAT: Technology-supported Task Oriented Arm Training) has been published in the conference proceedings of ICORR 2009 (International Conference of Rehabilitation Robotics, Kyoto, Japan).
- In a second sub-study the practical applicability of the ULTRA technology in assisting training of arm-hand functional performance in persons after stroke has been assessed. Furthermore, this pilot study yielded basic information as to possible benefit of the training regarding arm-hand skilled performance in persons after stroke that has been used for the power calculation of the third sub-study, i.e. the present RCT. Results of sub-study 2 will have been presented at the World Congress on Neurorehabilitation in Brazil in September 2008. An abstract has been published in Journal of Neurorehabilitation and Neural Repair (2008, 22; p.582). The full study report has been submitted to IEEE Transactions on Neural Systems and Rehabilitation Engineering.

<u>Research questions of the present RCT study</u>:

1) Does an 8 week technology-assisted task-oriented training program improve arm-hand function/activity and quality of life in persons who are in a chronic phase after stroke?

2) Does an 8 week technology-assisted task-oriented training program improve arm- hand function/activity and quality of life in persons who are in a chronic phase after stroke relative to task oriented training that is not supported by technology?

3) Does the treatment effect of task oriented training lasts longer after technology supported task oriented training relative to task oriented training without technology support in persons who are in a chronic stage after stroke?

4) Is treatment compliance during an 8 week technology-assisted task-oriented training program higher than during an 8 week task-oriented training program without technology support in persons who are in chronic phase after a stroke?

5) Are treatment credibility/expectancy and patient motivation higher in persons after stroke who train with technology versus persons after stroke who do not train with technology?

2. Content and plan of action for the RCT study

Characteristics of the participants
The participants are persons in the chronic phase after stroke. Persons in a chronic stage after stroke have obtained a (sub-optimal) functional level and generally demonstrate fewer fluctuations in motor performance. In the EXCITE trial [1], there were still significant improvements in arm hand function in the control group (3-9 months poststroke) even though no therapy or therapist attention was received. This means that up to 12 months a pre-entry plateau was not reached. Participants more than 1 year after stroke are also less influenced by different therapy approaches (physical therapy, occupational therapy, etc.) than persons in the sub-acute phase after stroke. Expected training effects will be smaller, but when present, are very meaningful. Disadvantages are that participant recruitment may be more difficult.

Inclusion criteria:

- first ever stroke
- 18-85 years old
- clinically diagnosed as having a central paresis of the arm/hand (strength Medical Research Council grade 2-3-4 at entry into the study)
- post-stroke time >=12 months
- fair-good cognitive level: MMSE (score above 26) [2]
- ability to read
- ability to understand Dutch language
- persons have to be impaired for at least two of the following skills: drinking from a cup, eating with knife and fork, taking money from purse, balancing object while holding it.
- Motivated to train on above mentioned skills

Exclusion criteria:

- severe neglect:
  - From medical files persons are selected that perform well on the O-zoek test: difference between omissions right and left >= 2 will not be included [3]. This test is not validated, but is used in Adelante and checking on its result will help us contacting persons that are appropriate to be included in this study.
  - Letter cancellation test and bell's test (only part of quantitative evaluation) to ensure that no severe neglect is present [4] [5]. These tests assess neglect in near extrapersonal space [6]. The omission score is calculated. A minimum omission score of 15% is considered to indicate unilateral spatial neglect (USN) in cancellation testing [7] [4]. Ferber and Karnath (2001) found that each of the latter tests only miss 6% of the neglect patients which is acceptable for this study [7].
- hemianopsia: retrieved from medical patient file
- severe spasticity: Modified Ashworth Scale total arm >4
- severe additional neurological, orthopaedic or rheumatoid impairments prior to stroke that may interfere with task performance
- Broca aphasia, Wernicke aphasia, global aphasia: as determined by Akense Afasie Test (AAT) (information obtained from medical files)
- severe apraxia: apraxietest van Heugten (information from medical files)

Participant Recruitment

Participant recruitment will happen at Adelante. The name of potential participants will be selected from the patient databases of Adelante by drs. W. Bakx and drs. M. Moennekens (rehabilitation specialists), based on the in- and exclusion criteria. Potential participants will be contacted by letter by drs. W.Bakx and drs. M. Moennekens. They will be able to indicate their willingness to participate by sending back, within 7 days, the willingness statement ('bereidheidverklaring'). After having stated their willingness to participate, the persons are contacted by phone by the researcher in order to make an appointment. During the first visit participants are given extensive information about the study and the procedures and tasks. They will be asked to sign an informed consent form. Subsequently they will enter the study as described in the protocol. Participants are free to ask any question related to the project, and answers will be provided at any time.

For participants that are selected for the study, letters to ask for participation in the study as well as participant information will be distributed by main researcher (Drs. A. Timmermans). Respondents will be protected by confidentiality and anonymity. All documents and data will be coded, using a combination of letter and number codes. The key to decode will be held by Dr. H. Seelen. Information that will be obtained will be used for research purposes only. Only group data will be reported. Four years after completion of the study, all data will be destroyed.

On their first visit, participants will be shown the Haptic Master system and will be informed about the training. After having given opportunity to answer all questions from the participant, an informed consent from the person for participation in the study is obtained. The participant is then educated in task-oriented training principles and the importance of frequent training to enlarge therapy success. Subsequently, baseline measurements will be performed.

Group size

The task-oriented training concept with Haptic Master is quite unique and clinical results of such training are not available for comparison with the current study, nor for use in a power analysis. A number of twenty two participants will be included in this study.

Randomisation procedure

Influx of participants into either control or experimental group will be based on computerized block randomization (block size=4).

Subjects experience varying levels of physical/occupational therapy and varying levels of exercise activity which are also distributed ad random over experimental and control group. Subjects are instructed not to change their routines during the 8 (2 + 6) months that they are participating in the study.

There is reason to expect that persons, whose dominant hand is impaired, will have a better training outcome than participants whose non-dominant hand is impaired. This is supported by findings of McCombe et al 2005 who found that patients with left hemispheric lesions have a better training response after 6 weeks of bilateral arm training than patients with right hemispheric lesions. Therefore a pre-stratification will occur for participants whose dominant side is impaired, in order to obtain an equal distribution of those participants over control and experimental group.

Participants are requested not to change their routines during the duration of the trial (8 months).

Study Design

The TEST-TRACS study is a mono-center, single-blinded, randomized controlled clinical trial

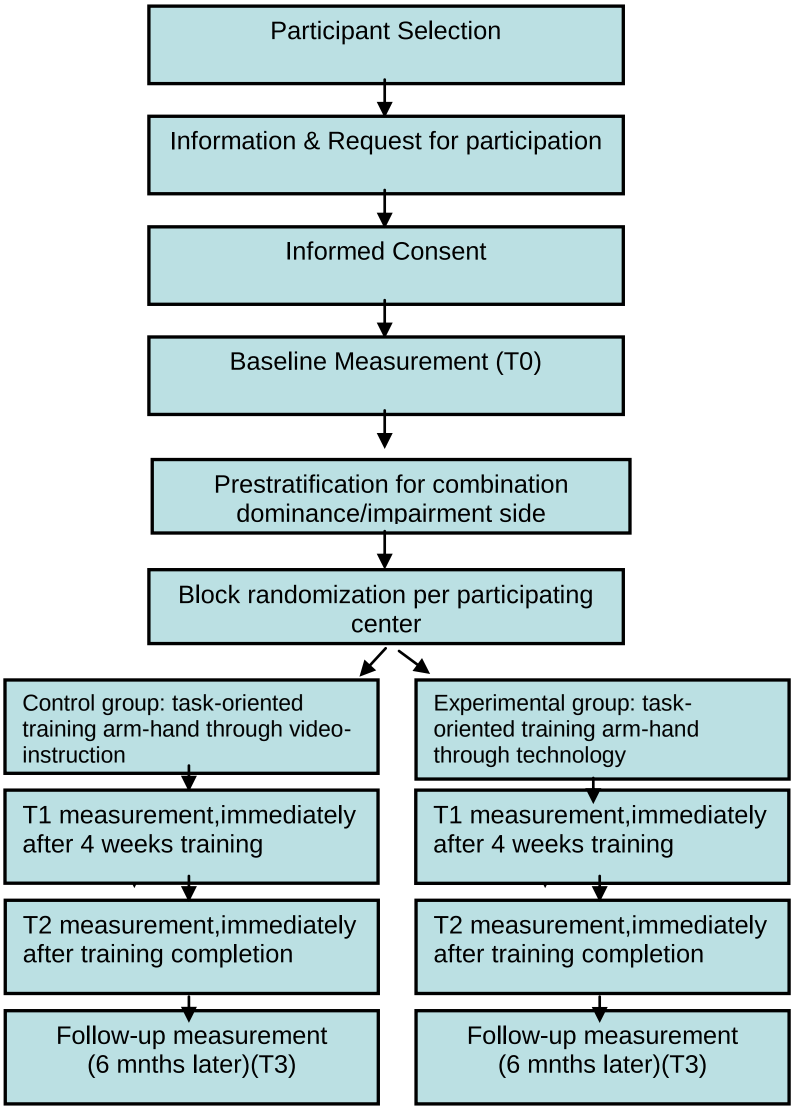

Choice of test instruments

Measurement tools for arm-hand function/activity:

Inter-individual differences in the stroke population, such as aetiology, location and severity of stroke make the selection of appropriate outcome measures a rather difficult task [8]. Several papers advice to choose outcome measures that assess arm and hand function so that impairment level, activity level, and participation level can be evaluated [9] [8] [10] [11] [12]. Functional scales that aim to assess improvement in the above parameters best serve as secondary outcome measures[13]. Our choice of scales is based on psychometric properties as well as them being used with the stroke population, as condition may influence sensitivity to change [8].

- *Kinematic parameter information from SAT(UM, dept. of movement sciences, Dr. J.J. Adam) such as reaction time, speed of movement and spatial error* are continuous variables that are excellent to use as primary outcome measures for analysis of

results in a task-oriented RCT [13]. The assessment method uses technology-support. The SAT-software program can be installed on any laptop or PC. The patient will be offered dots of 3 different sizes in random order. The dots appear on a touch screen that is mounted in the table surface in front of the patient. He/she should move his/her hand as fast and accurate as possible from one to the next target. The data that are collected via this application will give insights in central motor program changes after training and can shed light on fundamental neurophysiological processes of motor learning. Measurement tools with continuous outcomes are more likely to reflect gains than functional activity scales that do not employ ratio or interval measures [14]. Previously only one RCT has used kinematic analysis to investigate motor control strategies after task oriented training (CIMT training). The SAT test takes about 5 minutes to complete.

- *Fugl-Meyer Motor Assessment (FMMA)*.
  The FMMA consists of various separate sections. For this study only the section assessing motor function of the arm, wrist and hand is used. FMMA has been found a reliable (intra- and interrater reliability) and valid test for assessment of arm hand function in stroke patients [15] [16] [17] on impairment level [8] [10] [15]. The FMMA has been used as a gold standard against which other scales have been validated [10].
- *Action Research Arm Test (ARA(T)).*
  The ARA(T) has been proven to be a reliable, valid and sensitive instrument for upper limb function measurement, which does not show a ceiling effect [18] [19] [20] [21] [22].
- *Motor Activity Log (MAL), Dutch version*.
  The Motor Activity Log is a semi-structured interview to assess frequency of use and quality of use of the affected limb for skill performance. The MAL has been proven to be a reliable and valid tool for measurement of arm hand activity in stroke patients [23]. Dromerick et al [24] showed that patients with near perfect scores on ARA(T) show residual disability on MAL. Motor Activity Log Assessment will enable analysis of progress on the 2 skills that are trained in this study and possible transfer effects to other skills. Motor Activity Log is a self-evaluation scale; and therefore patients may have high expectations for self-appraisal because of the work they have put into training [14]. Another point to raise is that the MAL is evaluating items that have been trained; and is therefore likely to show better outcome measures [14].
- *Goal Attainment Scaling:GAS i*s a technique that evaluates the effectiveness of a treatment program on the basis of the extend to which patients achieve their individually set goals, as established on baseline measurement. GAS has been shown to be a reliable and valid method of outcome evaluation, responsive to change [25, 26]

Measurement tools for quality of life:

- *EuroQol-6D (EQ-6D*).
  This is a broad generic assessment based on a brief (3min) and simple questionnaire (either self-administered or completed on interview) [12] with acceptable test-retest reliability[27] and construct-validity [28]. Limitation is that EuroQol is more suitable for studying and comparing groups than for serial assessment of individual subjects [27]. Because EuroQol uses only few levels to rate QOL, any detected change in the domains over time is potentially important [27].
- *Medical Outcomes Study Short Form 36 (SF-36)(RAND36).*
  The SF-36 is a generic health survey to assess health status in the general population. It can be self-completed or can be administered by a trained interviewer [12]. It takes about 10 minutes to complete and has an acceptable test-retest reliability [27]. There is an indication that, in elderly persons, the SF-36 is more sensitive to changes than the EuroQol [29] . There has to be a difference of five points on the assessment over time, to be considered an important difference [27].

Measurement of therapy compliance:
In both, control group and experimental group, participants will keep a diary in which they mention exercise date, time and duration. Also it should be mentioned which exercises were practised.

Measurement of system usability
Two questions are rated on a VAS scale: 1) How good did you manage to use the system? And 2) Did you find the exercises challenging enough? A line is marked and subsequently measured in order for the result to obtain a score between 0 and 10.

Measurement of treatment credibility
In order to assess treatment credibility and subject's expectancy the credibility/expectancy questionnaire (CEQ) [30] will be administered directly following the explanation of the study's rationale to the participants, i.e. after informed consent has been obtained.

Measurement of cognitive functioning:
The Cognitive Log (CogLog) is a 10 item test that will be used to assess higher neurocognitive processes including: executive skills, attention, working memory, concentration and orientation. The test has been shown to be reliable and valid for usage with brain injured patients [31].

Measurement of patient motivation in general:
-The Health-Care-Self-Determination-Theory-Questionnaire (HCSDT) is a measure to assess motivation, based on the self-determination theory.
The self-determination theory evaluates the association between relationship-centered care and patients' motivation, behavior, family dynamics, health, and well-being. Three different needs are proposed by the self-determination theory: 1) the need for autonomy, 2) the need for competence and 3) the need for relatedness and support.
The HCSDT is an aggregate of three questionnaires. Questions are answered choosing the best fitting answer on a 7-point Likert rating scale. The HCSDT comprises of :a) the Treatment Self-Regulation Questionnaire (TSRQ) that has three subscales that address 'autonomous regulatory style' ('*autonomy*'); the 'controlled regulatory style' *('control'*) and '*a-motivation*'. An autonomous regulatory style is associated with higher internal (intrinsic) motivation, whereas a controlled regulatory style implies that individuals are driven by external rewards or forces. The a-motivation end of the continuum is associated with a complete absence of motivation, b) the Perceived Competence Scale (PCS) assesses the confidence patients have in their abilities and skills to do the things they choose ('*Competence*'), and c) the Health Care Climate Questionnaire (HCCQ short form) addresses patients' perception of the extent to which therapists are found to be supportive ('*Support*').

Measurement of patient motivation with regard to the specific training system:

-The intrinsic motivation inventory questionnaire (IMI) (Deci 1994, McAuley 1987) assesses participants' subjective experience related to a target activity. The instrument assesses the participants' interest/enjoyment, perceived competence, effort, value/usefulness, felt pressure and tension, and perceived choice while performing a given activity, thus yielding six subscale scores. A seventh subscale has been added to evaluate experiences of relatedness but validity of this subscale has not yet been established.

- A tool is being developed by Eindhoven University of Technology (Dept Industrial Design) in collaboration with Adelante to measure by means of *accelerometers* how much the affected arm is used in everyday life activities (with regard to the non-affected arm). The

accelerometers are incorporated in watch-like systems that can be worn on the right and left wrist. Feedback is given to the patients/therapists about the use of their affected arm in every day life situations. The feedback can be used for assessment of use of the affected arm in real life situations. The information reflects the importance of the changes after training for the every day life situation at home. The feedback can also be used to motivate patients for using the affected arm more in everyday life. The latter aspects underpin the relation between the tool developed and the other aims of the current project. However, before the system can be used for clinical trials, system usability and patient motivation have to be evaluated. For this purpose, the first 6 patients of the control group will be asked if they wish to attend (in the time that they are waiting between the two exercise sessions) this evaluation session, which will take approximately 30 minutes. This number of participants is generally accepted to be sufficient for the evaluation of system usability and patient motivation for system use [32]. The system will be shown to the participants, after which they will be asked to answer the questions of following questionnaires: IMI (duration 15 minutes), EQ-HQ-APPEAL (10 minutes) and CEQ (5 minutes). IMI and CEQ are explained below as they will also be used with regard to the Haptic Master use. EQ-HQ-APPEAL is a usability measure. Usability measures have traditionally been used to measure task-related efficiency and effectiveness of a product as a professional tool. Products that are easy to use may also become boring to use. The influence of perceived fun/enjoyment of a product next to ease of use is neglected in these studies. The EQ – HQ – APPEAL questionnaire is used to measure usability that incorporates key factors for designing appealing, enjoyable interfaces and systems. As well perceived quality aspects of the product (ergonomic quality, EQ, hedonic quality, HQ) as a final evaluation of the product are measured (APPEAL). Appeal is a weighted measure that people construct internally of the independent quality aspects.

Krebs et al [33] advocate that always the same therapist does the assessment of a certain scale, as variability in results between two experienced physiotherapists can be up to 15%, If not possible, it is advocated to train the therapists together intensively to guarantee uniformity of procedures and inter-rater reliability. The professional charged with assessment, should not be involved in therapy as he/she should not know who is enrolled in the experimental or control group (single-blind study).

**ICF Domain**	**Outcome Measure** (=OM)	**Test**	**T0**	**T1**	**T2**	**T3**
Activity Domain	Primary OM	Kinematic Parameter Info from SAT	x	x	x	x
Function Domain	Primary OM	Fugl Meyer	x	x	x	x
Activity Domain	Primary OM	ARA(T)	x	x	x	x
Activity Domain	Primary OM	Motor Activity Log	x	x	x	x
Activity Domain	Primary OM	Goal Attainment Scaling	x	x	x	x
Participation Domain	Secondary OM	EurQol-6D	x		x	x
Participation Domain	Secondary OM	SF-36 (RAND-36)	x		x	x
-	-	CEQ	x			
		SAT	x	x	x	x
		HCSDT<br>IMI<br>EQ-HQ-Appeal **	X<br><br>X		 x 	
-	Secondary OM	Therapy Compliance (diary)	x	x	x	
-	Secondary OM + Exclusion Measure	CogLog	x			
-	Exclusion Measure	Neglect Testing: Bells Test	x			
Function Domain	Exclusion Measure	Modified Ashworth Scale	x			

** = This questionnaire will only be used in 6 participants from the control group.

<u>The Haptic Master System</u>

Haptic-Master (Moog FCS BV, Nieuw-Vennep,The Netherlands) is a commercially available end-effector based robot that permits training of real-life functional tasks involving reach, grasp, as well as object transportation in three dimensional space. The environment is set-up to support seated as well as standing up task performance. The Haptic-Master is a six degrees of freedom (DoF) robot. Three active DoF's are for positioning and three passive DoF's are for orientation in the gimbal. This configuration permits the person to freely orient their hand as needed to manipulate an object. A six-axis force sensor located in the robot arm measures interaction forces (3 DoF) generated during tasks.
A software programme, named Haptic TOAT has been developed in Adelante (in cooperation with Hogeschool Zuyd) [34]. First, the movement-trajectory of the patient is recorded, with or without corrective therapeutic assistance. This record provides a base for training in a (1) passive movement mode and an (2) active movement mode. In a passive movement mode the HM takes the patient's arm along the recorded path. In an active movement mode patients were able to move through the recorded trajectory using their own skills and strengths. Patients have unlimited time to finish their task. This mode provided a unidirectional movement, where the amount of deviation could be controlled by changing spring-damper coefficients. The Haptic interface stays passive until the user deviates from the predefined path. In this case, the spring-damper combination encourages the patient to return to previously recorded pathway.

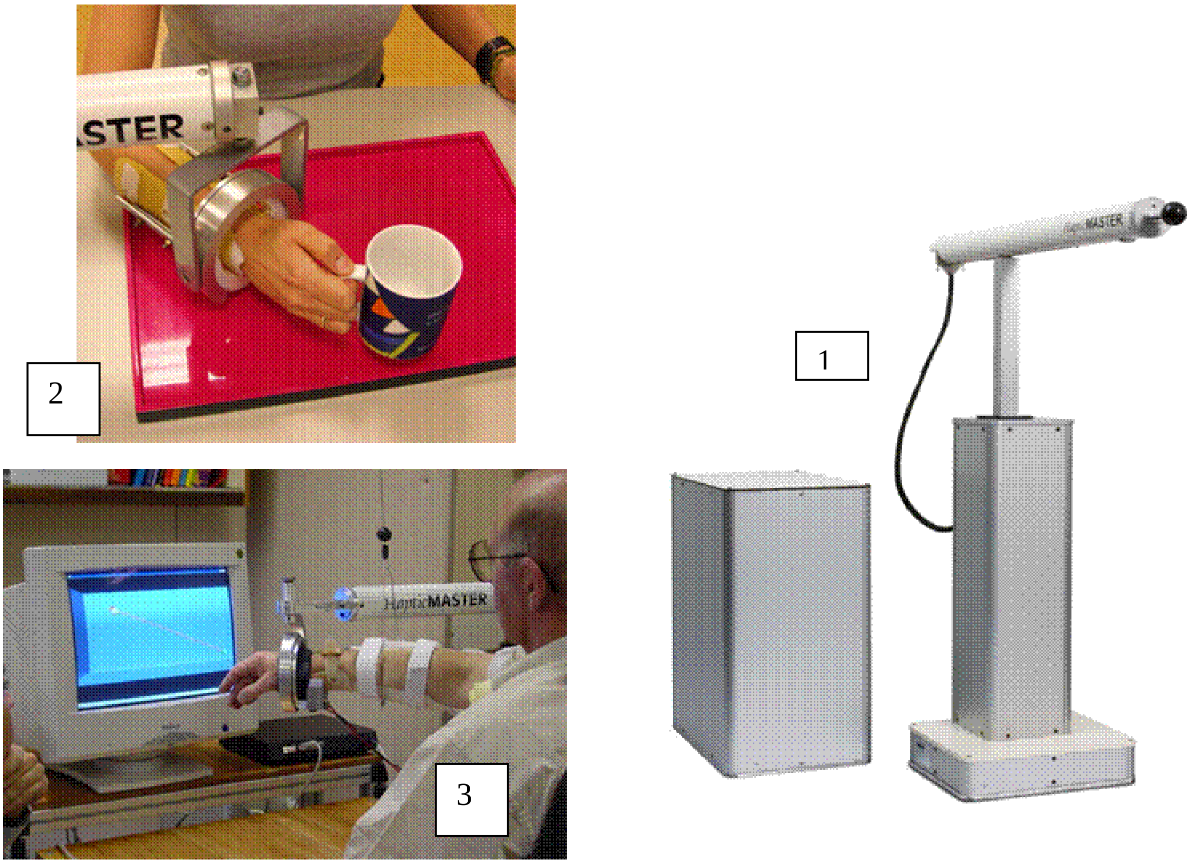

Figure: 1) The Haptic Master System, 2) Task-oriented training with Haptic Master and 3) Training with Haptic Master through games

Measurement time scale

Measurements: Measurement dates are: at the entry into the study (T0)(baseline); 4 weeks after T0 (T1); 8 weeks after T0 (T2) (training outcome), 6 months after T2 (T3)(long-term effects of training).

Inclusietijd (9 maanden) T0	Training (4 weeks) T1	Training (8 weeks) T2	Follow-up (6 months) T3

The assessment will always be performed by the same rehabilitation professional, who is not involved in the therapy.

Choice of skills to be trained

Training protocols for the following skills will be implemented: *'drinking from a cup'*, *'eating with knife and fork',' taking money from purse',' balancing object while holding it'.* The choice of skills is based on results from the skill preference study (sub-study 1). For each of the above-mentioned skills, training protocols have been elaborated. First the skills are broken

down into meaningful components that can be trained separately. For each of those components a training protocol is developed that offers engaging, challenging training (=increasing in exercise difficulty) and exercise variability. The training protocols have been implemented in Haptic Master to train the participants.

3. Training phase

The participants from the control group and experimental group are educated as to task-oriented training principles and the importance of frequent training to enlarge therapy success.
Participants in both groups train on at least two skills they select from a list of 4 skills established in a prior study (sub-study 1). All necessary materials for participation in the study (incl. DVD player or Haptic Master system), will be provided by the TEST-TRACS project.

The participants from the **control group** will receive videotaped instructions on a DVD from the task- oriented exercises that they have to do; together with the recommended performance frequency. These exercises are the same exercises as the ones the experimental group will do. Some minor adaptations were made to compensate for the absence of technology. The control intervention was tailored to a likely need of the subjects and reach toward the focus of the primary outcome measure [14]. All training happens in Adelante and is supervised by a therapist in the room.This way, participants in control and experimental group get the same amount of therapist attention.
If participants from the control group request to receive technology-supported training, then this will be offered after completion of the T3 measurement.

All participants from the **experimental group** will receive the same video-instructions of task-oriented training on a DVD but with the support of Haptic Master technology. After baseline measurements are performed, the participants will be familiarised with the Haptic Master use during the first session. All training happens in Adelante and is supervised by a therapist. The intervention aims to determine whether the Haptic Master system has an additional value over task oriented training without technology, not at finding out which component of the offered therapy dominates in causing a treatment effect.

According to Taylor et al, a typical training program after stroke comprises 4 to 8 exercises, 1 to 3 sets of 6 to 10 repetitions (load 10RM). Participants train 2 to 5 times per week for periods ranging from 4 to 12 weeks [35] .
All participants in the TEST-TRACS study (experimental and control group) will be asked to practise for 4 days per week during 8 weeks. Each day they log in for the training session for a minimum of 2 times 30 minutes (with a minimum one-hour break between both sessions). This means that subjects will train at least 4 hours (2 x ½ x 4) per week. There is an opportunity to exercise more if participants wish to do so, this from week 2 onwards (home training). If participants do not reach their prescribed training sessions during a week, these will be registered as non-compliance. The participant doesn't get a chance of prolonging the trial time (i.e. adding the lost days at the
end of the eight week period).
Dobkin [14] and Wolf et al [36]mention that it is essential that the control group should be matched to the experimental group with regard to intensity of practice. In our study the advocated minimum intensity of practice is the same for experimental and control group; although both groups are allowed to practice more if they wish to do so. This is the case, because it is assumed that the strength of technology supported training will be in the compelling to practice.

Participants will be compensated for travel costs to the medical center (0.19E per kilometer). This is important to avoid bias on participant selection with a profound sense of dedication; and also not considered a luxury when considering how participant recruitment is a major obstacle in organizing a RCT [36].
Patients can stop participating in the study at any moment without implications for further treatment. All data that have been collected until then will be destroyed.
All information and data from the study will be anonymized immediately. The key for decoding information will be kept by Dr. H. Seelen.
Anonymized data are available for scientific evaluation for Drs. A. Timmermans, Jolene van den Borne (Medical Doctor, assistant of Drs. M.Moennekens) and Maurice Monfrance (medical student University Maastricht) (researchers), Dr. H. Seelen (project leader and scientific research coordinator Adelante), Drs. W. Bakx en Drs. M. Moennekens (specialized medical doctors Adelante), Prof. Dr. H. Kingma (Technical University Eindhoven). Four years after completion of the study, all data will be destroyed.
Anonymized average group results will be reported in scientific context.

In case participants have questions (or complaints) that they prefer to discuss with somebody who is not a project member, they are offered to contact Mrs. Dr. Y. Janssen-Potten, program leader and senior researcher in Adelane, center of expertise in rehabilitation and audiology (045-5282373). If any advice is requested from an independent medical doctor, participants from the study can contact drs. J. Cluitmans, specialist in rehabilitation medicine in Adelante (045-5282322).

4. Data analysis

Average scores and SD scores of the kinematic data, the Fugl-Meyer test data and ARA(T) data will be calculated for T0, T1, T2 and T3. Statistical analysis of these data will include repeated measurement AN(C)OVA (multiple comparison), regression analysis, GEE**.**
The data of MAL, SF-36, EuroQol and other questionnaires will be reported descriptively.

5. Preparation of scientific report

The results of this sub-study will be reported in scientific journal papers and during international congresses.

6. Timeline

	08	09	09	09	09	09	09	09	09	09	09	09	09	10	10	10	10	10	10	10	10	10	10	10	10
	may	jan	feb	mar	apr	may	june	july	aug	sept	okt	nov	dec	jan	feb	mar	apr	may	juni	july	aug	sept	okt	nov	dec
METC proced.	**X**																								
Training therap.		**X**	**X**																						
Pat. Identific.				**X**	**X**	**X**	**X**	**X**	**X**	**X**	**X**														
Pat. inclusion						**X**	**X**			**X**	**X**	**X**	**X**	**X**	**X**	**X**									
Data coll.						**X**	**X**				**X**	**X**	**X**	**X**	**X**	**X**	**X**	**X**	**X**	**X**	**X**	**X**			
Data analysis																	**X**	**X**	**X**	**X**	**X**	**X**	**X**		
Report																							**X**	**X**	**X**

7. Partners in the project

AIO: Drs. Annick A.A. Timmermans, PT, MSc.

Medical student: Maurice Monfrance

Trainee in Rehabilitation Medicine: Jolene Van den Borne

Promotor: Prof. Dr. Herman Kingma (University Hospital Maastricht, Technical University Eindhoven).

Co-promotor: Dr. Henk Seelen (Adelante, Centre of Expertise in Rehabilitation and Audiology, Hoensbroek)

Project co-ordination:
Dr. Henk Seelen (Scientific research co-ordinator, Adelante Centre of Expertise in Rehabilitation and Audiology; Senior Researcher in University Maastricht, Maastricht School of Health, Department of Rehabilitation Medicine).

Project advisors for clinical aspects (Adelante/Hoensbroek):
Drs. Wip Bakx (physiatrist, Adelante),
Han Franck (occupational therapist, Adelante)
Paul Dobbelsteijn (physiotherapist, Adelante)

Collaborating Institutes:
Philips Research Eindhoven,
Adelante, Centre of Expertise in Rehabilitation and Audiology
Technical University Eindhoven

Project type: PhD research project

Budget: The project is fully funded by Philips Research Eindhoven, via the Technical University Eindhoven.

8. Literature

[1] S. L. Wolf, C. J. Winstein, J. P. Miller, E. Taub, G. Uswatte, D. Morris, C. Giuliani, K. E. Light, and D. Nichols-Larsen, "Effect of constraint-induced movement therapy on upper extremity function 3 to 9 months after stroke: the EXCITE randomized clinical trial," *Jama*, vol. 296, pp. 2095-104, 2006.

[2] R. M. Crum, J. C. Anthony, S. S. Bassett, and M. F. Folstein, "Population-based norms for the Mini-Mental State Examination by age and educational level," *Jama*, vol. 269, pp. 2386-91, 1993.

[3] C. H. Heugten van, K ; Sande van de ,P, "Cognitieve screening bij CVA-patienten in de revalidatie: normgegevens voor de klinische praktijk," *Tijdschrift Gerontologie en Geriatrie*, vol. 35, pp. 196-202, 2004.

[4] L. D. Gauthier, F ; Joanette,Y, "The Bells Test: A Quantitative and Qualitatitive Test For Visual Neglect," *International Journal of Clinical Neuropsychology*, vol. XI, pp. 49-54, 1989.

[5] B. Uttl and C. Pilkenton-Taylor, "Letter cancellation performance across the adult life span," *Clin Neuropsychol*, vol. 15, pp. 521-30, 2001.

[6] A. Menon and N. Korner-Bitensky, "Evaluating unilateral spatial neglect post stroke: working your way through the maze of assessment choices," *Top Stroke Rehabil*, vol. 11, pp. 41-66, 2004.

[7] S. Ferber and H. O. Karnath, "How to assess spatial neglect--line bisection or cancellation tasks?," *J Clin Exp Neuropsychol*, vol. 23, pp. 599-607, 2001.

[8] S. Barak and P. W. Duncan, "Issues in selecting outcome measures to assess functional recovery after stroke," *NeuroRx*, vol. 3, pp. 505-24, 2006.

[9] S. Geyh, A. Cieza, J. Schouten, H. Dickson, P. Frommelt, Z. Omar, N. Kostanjsek, H. Ring, and G. Stucki, "ICF Core Sets for stroke," *J Rehabil Med*, pp. 135-41, 2004.

[10] K. Salter, J. W. Jutai, R. Teasell, N. C. Foley, and J. Bitensky, "Issues for selection of outcome measures in stroke rehabilitation: ICF Body Functions," *Disabil Rehabil*, vol. 27, pp. 191-207, 2005.

[11] K. Salter, J. W. Jutai, R. Teasell, N. C. Foley, J. Bitensky, and M. Bayley, "Issues for selection of outcome measures in stroke rehabilitation: ICF activity," *Disabil Rehabil*, vol. 27, pp. 315-40, 2005.

[12] K. Salter, J. W. Jutai, R. Teasell, N. C. Foley, J. Bitensky, and M. Bayley, "Issues for selection of outcome measures in stroke rehabilitation: ICF Participation," *Disabil Rehabil*, vol. 27, pp. 507-28, 2005.

[13] B. H. Dobkin, "Interpreting the randomized clinical trial of constraint-induced movement therapy," *Arch Neurol*, vol. 64, pp. 336-8, 2007.

[14] B. H. Dobkin, "Confounders in rehabilitation trials of task-oriented training: lessons from the designs of the EXCITE and SCILT multicenter trials," *Neurorehabil Neural Repair*, vol. 21, pp. 3-13, 2007.

[15] D. J. Gladstone, C. J. Danells, and S. E. Black, "The fugl-meyer assessment of motor recovery after stroke: a critical review of its measurement properties," *Neurorehabil Neural Repair*, vol. 16, pp. 232-40, 2002.

[16] P. W. Duncan, M. Propst, and S. G. Nelson, "Reliability of the Fugl-Meyer assessment of sensorimotor recovery following cerebrovascular accident," *Phys Ther*, vol. 63, pp. 1606-10, 1983.

[17] J. Sanford, J. Moreland, L. R. Swanson, P. W. Stratford, and C. Gowland, "Reliability of the Fugl-Meyer assessment for testing motor performance in patients following stroke," *Phys Ther*, vol. 73, pp. 447-54, 1993.

[18] J. G. Broeks, G. J. Lankhorst, K. Rumping, and A. J. Prevo, "The long-term outcome of arm function after stroke: results of a follow-up study," *Disabil Rehabil*, vol. 21, pp. 357-64, 1999.
[19] R. C. Lyle, "A performance test for assessment of upper limb function in physical rehabilitation treatment and research," *Int J Rehabil Res*, vol. 4, pp. 483-92, 1981.
[20] C. L. Hsieh, I. P. Hsueh, F. M. Chiang, and P. H. Lin, "Inter-rater reliability and validity of the action research arm test in stroke patients," *Age Ageing*, vol. 27, pp. 107-13, 1998.
[21] W. H. M. Weerdt de, "Measuring recovery of arm-hand function in stroke patients: a comparison of the Brunnstrom-Fugl-Mayer test and the Action Research Arm test," *Physiotherapy Canada*, vol. 37, pp. 65-70, 1985.
[22] N. Yozbatiran, L. Der-Yeghiaian, and S. C. Cramer, "A standardized approach to performing the action research arm test," *Neurorehabil Neural Repair*, vol. 22, pp. 78-90, 2008.
[23] J. H. van der Lee, H. Beckerman, D. L. Knol, H. C. de Vet, and L. M. Bouter, "Clinimetric properties of the motor activity log for the assessment of arm use in hemiparetic patients," *Stroke*, vol. 35, pp. 1410-4, 2004.
[24] A. W. Dromerick, C. E. Lang, R. Birkenmeier, M. G. Hahn, S. A. Sahrmann, and D. F. Edwards, "Relationships between upper-limb functional limitation and self-reported disability 3 months after stroke," *J Rehabil Res Dev*, vol. 43, pp. 401-8, 2006.
[25] A. C. Reid, R, "Goal Attainment Scaling. Is it appropriate for stroke patients and their physiotherapists?," *Physiotherapy*, vol. 84, pp. 136-144, 1998.
[26] G. D. Bravo, MF. ; Roy,PM., "Improving the Quality of Residential Care Using Goal Attainment Scaling," *J Am Med Dir Assoc*, vol. 6, pp. 173-180, 2005.
[27] P. Dorman, J. Slattery, B. Farrell, M. Dennis, and P. Sandercock, "Qualitative comparison of the reliability of health status assessments with the EuroQol and SF-36 questionnaires after stroke. United Kingdom Collaborators in the International Stroke Trial," *Stroke*, vol. 29, pp. 63-8, 1998.
[28] N. Hoeymans, H. van Lindert, and G. P. Westert, "The health status of the Dutch population as assessed by the EQ-6D," *Qual Life Res*, vol. 14, pp. 655-63, 2005.
[29] J. E. Brazier, S. J. Walters, J. P. Nicholl, and B. Kohler, "Using the SF-36 and Euroqol on an elderly population," *Qual Life Res*, vol. 5, pp. 195-204, 1996.
[30] G. j. B. Devilly, T D, "Psychometric properties of the credibility/expectancy questionnaire," *Journal of Behavior Therapy and Experimental Psychiatry*, vol. 31, pp. 73-86, 2000.
[31] A. L. Alderson and T. A. Novack, "Reliable serial measurement of cognitive processes in rehabilitation: the Cognitive Log," *Arch Phys Med Rehabil*, vol. 84, pp. 668-72, 2003.
[32] J. Nielsen and T. Landauer, "A mathematical model of the finding of usability problems," presented at ACM INTERCHI'93 Conference Amsterdam, The Netherlands, 1993.
[33] H. I. Krebs, B. T. Volpe, M. Ferraro, S. Fasoli, J. Palazzolo, B. Rohrer, L. Edelstein, and N. Hogan, "Robot-aided neurorehabilitation: from evidence-based to science-based rehabilitation," *Top Stroke Rehabil*, vol. 8, pp. 54-70, 2002.
[34] A. A. A. Timmermans, R. P. J. Geers, J. A. Franck, P. Dobbelsteijn, A. I. F. Spooren, H. Kingma, and H. A. M. Seelen, "T-TOAT: A method of task-oriented arm training for stroke patients suitable for implementation of exercises in rehabilitation technology," presented at IEEE 11th International Conference on Rehabilitation Robotics, Kyoto International Conference Center, Japan, 2009.

[35] N. D. Taylor, KJ ; Damiano,DL, "Progressive Resistance Exercise in Physical Therapy: A Summary of Systematic Reviews," *Physical Therapy*, vol. 85, pp. 1208-1222, 2005.

[36] S. L. Wolf, C. J. Winstein, J. P. Miller, S. Blanton, P. C. Clark, and D. Nichols-Larsen, "Looking in the rear view mirror when conversing with back seat drivers: the EXCITE trial revisited," *Neurorehabil Neural Repair*, vol. 21, pp. 379-87, 2007.

Annex I: Letter to request subject participation in the study.

Hoensbroek, 5 oktober 2009

Kenmerk: Adelante/ AT-HS/01a

Betreft: Verzoek tot medewerking aan het onderzoek getiteld “TEST-TRACS: Studie naar het effect van technologie-ondersteunde arm- en handvaardigheidstraining bij mensen na een CVA”

Geachte heer, mevrouw,

Graag wil ik uw medewerking vragen voor een onderzoek dat uitgevoerd wordt in Adelante (voorheen: Stichting Revalidatie Limburg). De titel van het onderzoek luidt: “TEST-TRACS: Studie naar het effect van technologie-ondersteunde arm- en handvaardigheidstraining bij mensen na een CVA”. Dit onderzoek wordt uitgevoerd in samenwerking met Philips Research en de Technische Universiteit Eindhoven.

Momenteel wordt een systeem ontwikkeld dat mensen na een CVA zou kunnen begeleiden bij het ‘zelf oefenen’ van dagelijkse vaardigheden. Dit systeem heet Haptic Master.
In het onderzoek wordt nagegaan of mensen na een CVA die taakgericht trainen met het Haptic Master systeem meer vooruitgaan dan mensen die taakgericht trainen zonder het Haptic Master systeem. De training richt zich op arm- en handvaardigheid. Er wordt een keuze aangeboden voor het trainen uit volgende vaardigheden: eten met mes en vork; drinken uit een kop, geld uit beurs nemen, een object in evenwicht houden.
Aan dit onderzoek zullen 22 mensen deelnemen. Er wordt gedurende 8 weken getraind. Het trainingsprogramma is zo opgesteld dat wij hopen op een positief trainingseffect. Wij zijn er echter niet zeker van of deze behandeling ook daadwerkelijk effect heeft.

Ik heb, na zorgvuldige toetsing van het onderzoek door de Medische Ethische Toetsingscommissie van Adelante, het patiëntenbestand van afdeling Hersenletselrevalidatie van Adelante geraadpleegd. Hierbij is ook uw naam geselecteerd. In bijgaande brief vindt u meer informatie betreffende dit onderzoek. Als u geïnteresseerd bent in deelname aan het onderzoek, dan verzoek ik u bijgaande bereidheidverklaring te sturen naar het secretariaat van het Kenniscentrum van Adelante (door middel van bijgevoegde antwoordenveloppe). In verband met de planning van het onderzoek, verzoek ik u te reageren binnen 7 dagen.
Mocht u vragen hebben naar aanleiding van deze brief, dan kunt u contact opnemen met ondergetekende.
In afwachting van uw antwoord teken ik.
Met vriendelijke groet,

Drs. W. Bakx, Revalidatiearts
Adelante
Zandbergsweg 111
6432 CC Hoensbroek
tel: 045 5282340

Hoensbroek, 5 oktober 2009

Kenmerk: Adelante/ AT-HS/02a

Betreft: Verzoek tot medewerking aan het onderzoek getiteld "TEST-TRACS: Studie naar het effect van technologie-ondersteunde arm- en handvaardigheidstraining bij mensen na een CVA"

Geachte heer, mevrouw,

Graag wil ik uw medewerking vragen voor een onderzoek dat uitgevoerd wordt in Adelante (voorheen: Stichting Revalidatie Limburg). De titel van het onderzoek luidt: "TEST-TRACS: Studie naar het effect van technologie-ondersteunde arm- en handvaardigheidstraining bij mensen na een CVA". Dit onderzoek wordt uitgevoerd in samenwerking met Philips Research en de Technische Universiteit Eindhoven.

Momenteel wordt een systeem ontwikkeld dat mensen na een CVA zou kunnen begeleiden bij het 'zelf oefenen' van dagelijkse vaardigheden. Dit systeem heet Haptic Master.
In het onderzoek wordt nagegaan of mensen na een CVA die taakgericht trainen met het Haptic Master systeem meer vooruitgaan dan mensen die taakgericht trainen zonder het Haptic Master systeem. De training richt zich op arm- en handvaardigheid. Er wordt een keuze aangeboden voor het trainen uit volgende vaardigheden: eten met mes en vork; drinken uit een kop, geld uit beurs nemen, een object in evenwicht houden.
Aan dit onderzoek zullen 22 mensen deelnemen. Er wordt gedurende 8 weken getraind. Het trainingsprogramma is zo opgesteld dat wij hopen op een positief trainingseffect. Wij zijn er echter niet zeker van of deze behandeling ook daadwerkelijk effect heeft.

Ik heb, na zorgvuldige toetsing van het onderzoek door de Medische Ethische Toetsingscommissie van Adelante, het patiëntenbestand van afdeling Hersenletselrevalidatie van Adelante geraadpleegd. Hierbij is ook uw naam geselecteerd. In bijgaande brief vindt u meer informatie betreffende dit onderzoek. Als u geïnteresseerd bent in deelname aan het onderzoek, dan verzoek ik u bijgaande bereidheidverklaring te sturen naar het secretariaat van het Kenniscentrum van Adelante (door middel van bijgevoegde antwoordenveloppe). In verband met de planning van het onderzoek, verzoek ik u te reageren binnen 7 dagen.
Mocht u vragen hebben naar aanleiding van deze brief, dan kunt u contact opnemen met ondergetekende.
In afwachting van uw antwoord teken ik.
Met vriendelijke groet,

Met vriendelijke groet,

Drs. M. Moennekens, Revalidatiearts
Adelante
Zandbergsweg 111
6432 CC Hoensbroek
tel: 045 5282365

Annex II: Information for participants.
Informatiebrief voor deelnemers afdeling 4 en 5 aan onderzoek met de titel: “TEST-TRACS: Studie naar het effect van technologie-ondersteunde arm- en handvaardigheidstraining bij mensen na een CVA”.

Hoensbroek, 5 oktober 2009

Kenmerk: Adelante/ AT-HS/03a

Betreft: Informatiebrief voor het onderzoek getiteld: “TEST-TRACS: Studie naar het effect van technologie-ondersteunde arm- en handvaardigheidstraining bij mensen na een CVA”

Geachte heer, mevrouw,

Hierbij ontvangt u nadere informatie over het wetenschappelijk onderzoeksproject TEST-TRACS. In dit onderzoek willen we testen wat het effect is van systeemondersteuning bij het taakgericht trainen van arm-handfunctie.

Het onderzoek

Momenteel wordt een systeem ontwikkeld dat mensen na een CVA begeleidt bij het ‘zelf-oefenen’ van dagelijkse vaardigheden. Dit systeem heet Haptic Master. Het bestaat uit drie delen: 1) een computerscherm met daarop informatie over het bewegen, 2) een op maat gemaakt trainingsplan voor armen en handen, 3) een robotarm die uw arm kan helpen bij het bewegen. De ontwikkeling van dit systeem is nog in een beginstadium. Het is nog niet ‘in de winkel’ verkrijgbaar.

In het onderzoek wordt nagegaan of de arm-handvaardigheid van mensen meer dan 1 jaar na een CVA door taakgericht trainen nog verder kan verbeteren. Ook wordt nagegaan of trainen met het Haptic Master-systeem de arm-handvaardigheid meer verbetert dan taakgericht trainen zonder het Haptic Master-systeem.
Voor dit onderzoek zijn trainingsprogramma’s uitgewerkt in Adelante voor de volgende vaardigheden: eten met mes en vork; drinken uit een kop, geld uit een beurs nemen, een object in evenwicht houden.
Aan dit onderzoek nemen 22 mensen deel. De helft van de deelnemers aan het onderzoek zullen de trainigsprogramma’s uitvoeren op basis van DVD’s met oefeninstructies. De andere helft van de deelnemers aan het onderzoek krijgen dezelfde oefeninstructies aangereikt via het Haptic Master systeem. Door middel van loting wordt bepaald tot welke groep u zult behoren.
Indien, na afloop van de studie zou blijken dat de deelnemers van de Haptic Master groep meer vooruitgang in arm-handvaardigheid geboekt hebben, kunnen de deelnemers die het andere trainingsprogramma gevolgd hebben, indien zij dit zouden wensen, het Haptic Master trainingsprogramma doorlopen.

Waar, wanneer en wie?

Het onderzoek zal plaatsvinden in de maanden oktober 2009 tot en met september 2010.
Dit onderzoek wordt uitgevoerd in Adelante. De studie zal gebeuren in samenwerking met Philips Research en de Technische Universiteit Eindhoven.
De testafname en de trainingen worden begeleid door ergotherapeuten en fysiotherapeuten van Adelante. Het onderzoek wordt uitgevoerd door ondergetekende, drs. A. Timmermans (onderzoeker Adelante) met medewerking van drs. W. Bakx en drs. M. Moennekens (revalidatieartsen van de afdelingen hersenletselrevalidatie in Adelante). Dr. H. Seelen (onderzoekscoördinator Adelante) is projectleider van het onderzoek.

De reiskosten die u eventueel maakt ten behoeve van het onderzoek worden door ons vergoed aan een bedrag van 0.19 Euro per kilometer.

Waarom is dit onderzoek nu zo belangrijk?

Uit onderzoek, maar ook uit de dagelijkse revalidatiepraktijk blijkt dat bij veel mensen die een CVA gehad hebben het herstel van de arm- en handvaardigheid achterblijft.
De technologie die momenteel wordt ontwikkeld, probeert mensen met een CVA extra oefenmogelijkheden te geven, waarbij ze zelfstandig kunnen oefenen. De verwachting is dat arm-handvaardigheidstraining leidt tot een verbetering van arm-hand functie en dat er een verschil in effect optreedt tussen technologie-gebruik (Haptic Master) en DVD gebaseerde training. Deze studie onderzoekt de effecten van technologie-gestuurde arm-hand training. De resultaten kunnen bijdragen aan een verbetering van arm-handvaardigheidstraining bij mensen na een CVA in de toekomst.

Wie en wat?

Aan het onderzoek kunnen mensen deelnemen die meer dan 12 maanden geleden een beroerte gehad hebben. Het onderzoek bestaat uit een trainingsdeel en een meetdeel. Het totale onderzoek zal ongeveer 8 weken duren. Hierbij wordt van u verwacht dat u tijdens deze 8 weken minstens 4 dagen per week traint. Elk van deze dagen traint u tenminste 2x 30 minuten (met minimum een uurtje pauze tussen deze twee sessies). De training vindt plaats in Adelante in Hoensbroek zodat de behandelaar/therapeut die in de ruimte aanwezig is, op afstand kan meekijken/helpen en de oefeningen indien nodig kan bijsturen.
Op 4 momenten in de tijd worden metingen verricht bestaande uit vragenlijsten rond levenskwaliteit en korte arm- en handfunctietesten. Hierbij wordt van u gevraagd om bepaalde arm-handbewegingen uit te voeren, die door een ervaren therapeut beoordeeld worden. Ook wordt u gevraagd een korte vragenlijst in te vullen. Per keer duren de testen een tweetal uren. De meettijdstippen zijn voor het begin van de training, na 4 weken trainen, na 8 weken trainen en 6 maanden na het beëindigen van de training. Aan 6 van de 22 deelnemers zal gevraagd worden om drie korte extra vragenlijsten in te vullen. Hiermee wordt uw mening gevraagd over een bewegingsmeter (in de vorm van 2 polshorloges). Dit duurt ongeveer een half uur.
Wij zoeken personen die geïnteresseerd zijn in het trainen van 2 of meer van volgende vaardigheden: eten met mes en vork; drinken uit een kop, geld uit beurs nemen, een object in evenwicht houden. Wij zoeken personen die een verminderde arm hand functie hebben ten gevolge van een CVA, maar die wel enige spierkracht in de arm hebben. U mag niet teveel last hebben van spasticiteit.
Deelname aan het onderzoek houdt in dat u toestemming geeft aan uw revalidatiearts om voor dit onderzoek enkele gegevens uit de medische status te halen, te weten: leeftijd, geslacht, datum en lokalisatie van de beroerte, informatie over eventuele aanwezigheid van spasticiteit, uw gezichtsvermogen, uw spraakvermogen en uw cognitieve vaardigheden. U zal gevraagd worden om een toestemmingsformulier te ondertekenen bij aanvang van de studie.

Uw rechten en onze plichten

Uw medewerking aan het onderzoek is geheel vrijwillig. U hebt te allen tijde het recht om zonder opgaaf van reden deelneming aan het onderzoek te weigeren. Dit zal geen gevolgen hebben voor uw eventuele behandeling. Uw gegevens die tot dan verzameld zijn, zullen vernietigd worden. U hebt bovendien het recht om meer informatie over het onderzoek te vragen. U heeft inzage in de door ons geregistreerde gegevens en resultaten van uzelf. Alle gegevens en resultaten van het onderzoek worden anoniem en vertrouwelijk behandeld. Dit wil zeggen dat alle informatie direct gecodeerd wordt, en dat niet gecodeerde gegevens

(bijvoorbeeld uw naam) gescheiden opgeslagen wordt van bijvoorbeeld de testresultaten. De sleutel om de gegevens te decoderen wordt bewaard door Dr. H. Seelen.
De anoniem geregistreerde gegevens zijn ter inzage van Drs. A. Timmermans (onderzoeker), Dr. H. Seelen (onderzoekscoördinator Adelante en leider van dit project), Drs. W. Bakx en Drs. M. Moennekens (revalidatieartsen Adelante), Maurice Monfrance (geneeskundestudent Universiteit Maastricht), Jolene van den Borne (arts-assistent), Prof. Dr. H. Kingma (Hoogleraar aan de Technische Universiteit Eindhoven). Vier jaar na afloop van het onderzoek zullen alle identificeerbare gegevens worden vernietigd, zodat niemand meer kan nagaan welke gegevens bij welke personen horen.
De algemene resultaten van het onderzoek, dat wil zeggen de anonieme gemiddelde groepsresultaten, zullen beschreven worden in een wetenschappelijk rapport.

Veiligheid en verzekering
Het risico bij dit onderzoek is niet groter dan bij gewone dagelijkse activiteiten. Volgens de Wet Medisch Wetenschappelijk Onderzoek met Mensen die per 1 december 1999 in werking is getreden, dient in principe voor elk onderzoek waarbij proefpersonen betrokken zijn een verzekering te worden afgesloten. De Medisch Ethische Toetsingscommissie Adelante heeft het onderzoek getoetst en heeft een positief oordeel afgegeven. U zult tijdens het onderzoek verzekerd zijn volgens de proefpersonenverzekering van Adelante. In de bijlage vindt u meer informatie over deze verzekering.

Klachten of vragen?
Mocht u klachten of vragen hebben over de inhoud of het verloop van het onderzoek die u liever niet aan een projectmedewerker wilt voorleggen, dan kunt u contact opnemen met Mw. Dr. Y. Janssen-Potten, programmaleider en onderzoeker Adelante (045-5282373 op maandag en dinsdag).
Mocht u voor, tijdens, of na het onderzoek inlichtingen of advies willen van een onafhankelijke arts t.a.v. het onderzoek of het nut van het onderzoek voor uzelf, kunt u contact opnemen met drs. J. Cluitmans (045-5282322), revalidatiearts, welke niet betrokken is bij het onderzoek.

Bent U geïnteresseerd?
Door middel van deze brief willen wij nagaan of u geïnteresseerd bent om aan dit onderzoek mee te doen. Via bijgevoegd formulier kunt u kenbaar maken of u in principe bereid bent mee te doen aan dit onderzoek. U kunt dit formulier kosteloos in bijgevoegde briefomslag opsturen aan het secretariaat van de afdeling Wetenschappelijk Onderzoek, Adelante (t.a.v. A. Timmermans), Antwoordnummer 10005, 6400 VG Hoensbroek.
In verband met de planning van het onderzoek verzoeken wij u om binnen de 7 dagen te reageren. Als u te kennen hebt gegeven dat u bereid bent om deel te nemen aan het onderzoek, zal contact met u worden opgenomen voor verdere afspraken.
Mocht u vragen hebben naar aanleiding van deze brief, dan kunt u contact opnemen met ondergetekenden.

Met vriendelijke groet,

Drs. A.A.A. Timmermans
Onderzoeker,
Adelante Kenniscentrum
Zandbergsweg 111
6432 CC Hoensbroek

Tel. 045-5282372

Dr. H.A.M. Seelen
Coördinator Onderzoek
Adelante Kenniscentrum
Zandbergsweg 111
6432 CC Hoensbroek

tel. 045-5282221

ANNEX III: Verzekeringsbijlage

Verzekeringsbijlage Adelante bij informatiebrief

Voor de deelnemers aan dit onderzoek is door de Adelante een verzekering afgesloten. Deze verzekering dekt schade door dood of letsel die het gevolg is van deelname aan het onderzoek, en die zich gedurende de deelname aan het onderzoek openbaart, of binnen vier jaar na beëindiging van de deelname aan het onderzoek. De schade wordt geacht zich te hebben geopenbaard wanneer deze bij de verzekeraar is gemeld.

In geval van schade kunt u zich direct wenden tot de verzekeraar.
De verzekeraar van het onderzoek is:

Naam:	Onderlinge Waarborgmaatschappij Centramed B.A.
Adres:	Appelgaarde 4<br>Postbus 191<br>2270 AD Voorburg
Telefoonnummer:	070-3017070
Contactpersoon:	dhr. H. Pardoen

De verzekering biedt een maximum dekking van € 450.000 per proefpersoon en € 3.500.000 voor het gehele onderzoek, en € 5.000.000 per jaar voor alle onderzoeken van dezelfde opdrachtgever. De dekking van specifieke schades en kosten is verder tot bepaalde bedragen beperkt. Dit is opgenomen in het Besluit verplichte verzekering bij medisch-wetenschappelijk onderzoek met mensen. Informatie hierover kunt u vinden op de website van de Centrale Commissie Mensgebonden Onderzoek: www.ccmo.nl.

Voor deze verzekering gelden een aantal uitsluitingen.
De verzekering dekt niet:

- aanspraken voor schade die het gevolg is van het uitblijven van een vermindering van de gezondheidsproblemen van de proefpersoon, danwel het gevolg is van de verdere verslechtering van de gezondheidsproblemen van de proefpersoon, indien de deelname van de proefpersoon aan het wetenschappelijk onderzoek geschiedt in het kader van de behandeling van deze gezondheidsproblemen;
- aanspraken voor schade door aantasting van de gezondheid van de proefpersoon waarvan aannemelijk is dat die zich ook zou hebben geopenbaard wanneer de proefpersoon niet aan het onderzoek had deelgenomen;
- aanspraken voor schade door aantasting van de gezondheid van de proefpersoon in het geval deze deelneemt aan een vergelijkend onderzoek als bedoeld in artikel 4 tweede lid van de Algemene Maatregel van Bestuur en aannemelijk is dat de schade het gevolg is van de in dat lid bedoelde reeds toegepaste handeling waaraan de proefpersoon wordt onderworpen;
- aanspraken voor schade die zich openbaart bij nakomelingen als gevolg van een nadelige inwerking van medisch-wetenschappelijk onderzoek op de proefpersoon en/of de nakomeling;
- aanspraken voor schade waarvan het op grond van de aard van het medisch-wetenschappelijk onderzoek zeker of nagenoeg zeker is dat deze zich zou voordoen;
- aanspraken voor schade die het gevolg is van het niet of niet volledig opvolgen van aanwijzingen en instructies door de proefpersoon, voor zover de proefpersoon daartoe in staat is.

Annex IV: Bereidheidverklaring.

Bereidheidverklaring voor deelnemers aan het onderzoek met de titel: "TEST-TRACS: Studie naar het effect van technologieondersteunde arm- en handvaardigheidstraining bij mensen na een CVA"

Kenmerk: Adelante/ AT-HS/04a

Ondergetekende is in principe bereid deel te nemen aan het in de informatiebrief (kenmerk Adelante/ AT-HS/03a) beschreven onderzoek getiteld: :"TEST-TRACS: Studie naar het effect van technologieondersteunde arm- en handvaardigheidstraining bij mensen na een CVA"

Naam: ____

Adres: ____

Postcode: ____

Woonplaats: ____

Telefoonnummer: ____

Datum: ____

Handtekening deelnemer: ______________________________________

U kunt deze bereidheidverklaring in bijgevoegde antwoordenveloppe opsturen naar:
Adelante
t.a.v. Adelante Kenniscentrum (A. Timmermans)
Antwoordnummer 10005
6400 VG Hoensbroek

Annex V: Toestemmingsformulier

Toestemmingsformulier voor deelnemers aan het onderzoek met de titel: "TEST-TRACS: Studie naar het effect van technologieondersteunde arm- en handvaardigheidstraining bij mensen na een CVA"

Kenmerk: Adelante/ AT-HS/05a

Ik,
ondergetekende____
verklaar kennis te hebben genomen van de informatie, uitvoering en strekking van het onderzoek met de titel: "TEST-TRACS: Studie naar het effect van technologieondersteunde arm- en handvaardigheidstraining bij mensen na een CVA". Deze informatie heb ik ontvangen door middel van een informatiebrief (kenmerk: Adelante/ AT-HS/03a) en door middel van mondelinge uitleg. Ik begrijp de opzet en het doel van het onderzoek en ik weet wat er van mij verwacht wordt.

Ik ben tevens op de hoogte van de volgende punten:

- Het algemene doel van het onderzoek is te beoordelen welk het effect is van taakgericht trainen van arm- en handvaardigheid bij mensen na een CVA met en zonder technologie (in dit geval het Haptic Master systeem)
- Het onderzoek wordt uitgevoerd in Adelante te Hoensbroek in samenwerking met Philips Research en Technische Universiteit Eindhoven.
- Het onderzoek zal 8 weken duren.
- Tijdens de duur van het onderzoek train ik tenminste 4 dagen per week, voor tenminste 2x 30 minuten.
- Ik kan met het Haptic Master systeem of met een DVD met oefeninstructies trainen. Indien ik tot de groep behoor die traint met Haptic Master, wordt mijn onderarm door middel van een polssteun bevestigd aan de Haptic Master. Ik kan de Haptic Master op elk moment in de training zelf stoppen.
- Voordat het onderzoek start, zullen enkele korte arm- en handtesten bij mij worden afgenomen, evenals enkele vragenlijsten in verband met levenskwaliteit. De afname van testen en vragenlijsten zal herhaald worden na vier weken trainen, na 8 weken trainen en 6 maanden na het beëindigen van de training. De vragen rond levenskwaliteit worden afgenomen via een gestructureerd vraaggesprek. Bij de arm- en handtesten zal gevraagd worden om bepaalde eenvoudige bewegingen met armen en handen uit te voeren.
- Ik weet dat ik mogelijk gevraagd word om drie korte extra vragenlijsten in te vullen. Hiermee wordt mijn mening gevraagd over een bewegingsmeter (in de vorm van 2 polshorloges). Dit deel is eenmalig en duurt ongeveer een half uur.
- Deelname aan het onderzoek is vrijwillig. Ik verplicht mij tot niets. Terugtrekken uit het onderzoek is altijd mogelijk zonder opgaaf van reden en zonder dat het gevolgen heeft voor mijn (eventuele) behandeling.
- Ik kan op elk tijdstip voor, tijdens en na het onderzoek vragen stellen ten aanzien van het onderzoek. Aan het einde van het onderzoek zal informatie gegeven worden over de resultaten.
- De resultaten van het onderzoek zullen anoniem en vertrouwelijk behandeld worden.
- Ik geef toestemming dat de revalidatiearts de volgende gegevens uit mijn medisch dossier haalt ten behoeve van het onderzoek:
    - leeftijd
    - geslacht
    - datum en lokalisatie van de hersenbloeding

- informatie rond gezichtsvermogen en spraak
- eventuele aanwezigheid van spasticiteit
- cognitieve status

- Vier jaar na afloop van het onderzoek zullen alle identificeerbare gegevens vernietigd worden, zodat niemand meer kan nagaan welke gegevens bij welke personen horen.
- De anoniem geregistreerde gegevens zijn ter inzage beschikbaar voor drs. A. Timmermans (onderzoeker Adelante), dr. H. Seelen (onderzoekscoördinator Adelante en projectleider TEST-TRACS), drs. W. Bakx en drs. M. Moennekens (revalidatieartsen Adelante), J. van den Borne (revalidatiearts in opleiding) en M. Monfrance (student Geneeskunde).
- De risico's tijdens het onderzoek zijn niet groter dan bij gewone activiteiten in het dagelijks leven.
- Gedurende het onderzoek ben ik verzekerd via de proefpersonenverzekering van Adelante.
- De bij het onderzoek betrokken projectmedewerkers drs. M. Moennekens, drs. W. Bakx, dr. H. Seelen, M. Monfrance, J. van den Borne en drs. A. Timmermans zorgen ervoor dat het onderzoek uitgevoerd wordt volgens de verstrekte gegevens met inachtneming van privacy van de deelnemers.

Plaats:____

Datum:____

Handtekening deelnemer:____

Naam projectuitvoerder:____

Handtekening projectuitvoerder:____

N.B. Dit formulier is in tweevoud verstrekt. Een exemplaar is voor de projectuitvoerder, het andere exemplaar is voor de deelnemer bestemd.

ANNEX VI: VRAGENLIJSTEN

1. EuroQol Gezondheidsvragenlijst

(Nederlandse versie)

Zet bij iedere groep in de lijst hieronder een kruisje in het hokje achter de zin die het best past bij uw eigen gezondheidstoestand vandaag.

Mobiliteit

Ik heb geen problemen met lopen ❑

Ik heb enige problemen met lopen ❑

Ik ben bedlegerig ❑

Zelfzorg

Ik heb geen problemen om mijzelf te wassen of aan te kleden ❑

Ik heb enige problemen om mijzelf te wassen of aan te kleden ❑

Ik ben niet in staat mijzelf te wassen of aan te kleden ❑

Dagelijkse activiteiten *(bijv. werk, studie, huishouden, gezins- en vrijetijdsactiviteiten)*

Ik heb geen problemen met mijn dagelijkse activiteiten ❑

Ik heb enige problemen met mijn dagelijkse activiteiten ❑

Ik ben niet in staat mijn dagelijkse activiteiten uit te voeren ❑

Pijn/klachten

Ik heb geen pijn of andere klachten ❑

Ik heb matige pijn of andere klachten ❑

Ik heb zeer ernstige pijn of andere klachten ❑

Stemming

Ik ben niet angstig of somber ❑

Ik ben matig angstig of somber ❑

Ik ben erg angstig of somber ❑

Om mensen te helpen bij het aangeven hoe goed of hoe slecht een gezondheidstoestand is, hebben we een meetschaal (te vergelijken met een thermometer) gemaakt. Op de meetschaal hiernaast betekent "100" de beste gezondheidstoestand die u zich kunt voorstellen, en "0" de slechtste gezondheidstoestand die u zich kunt voorstellen.

We willen u vragen op deze meetschaal aan te geven hoe goed of hoe slecht volgens u uw eigen gezondheidstoestand vandaag is. Trek een lijn van het hokje hieronder naar het punt op de meetschaal dat volgens u aangeeft hoe goed of hoe slecht uw gezondheidstoestand vandaag is.

Uw gezondheidstoestand vandaag

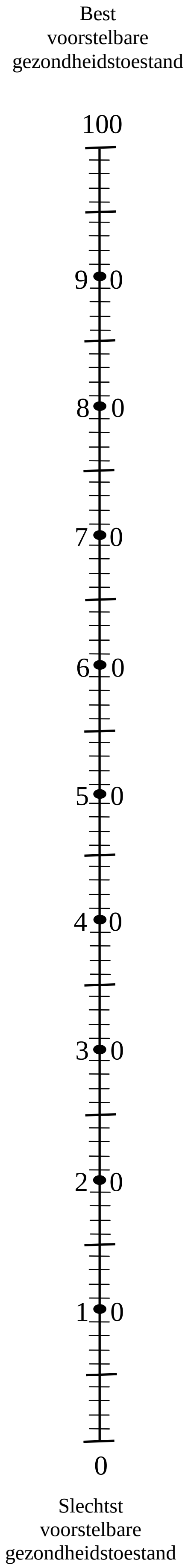

2. SF-36 Gezondheidstoestand Vragenlijst

Instructie: Deze vragenlijst gaat over uw standpunten t.a.v. uw gezondheid. Met behulp van deze gegevens kan worden bijgehouden hoe U zich voelt en hoe goed U in staat bent Uw gebruikelijke bezigheden uit te voeren.

Beantwoord elke vraag door het antwoord op de aangegeven wijze te markeren. Als U niet zeker weet hoe U een vraag moet beantwoorden, geef dan het best mogelijke antwoord.

1. **Hoe zou U over het algemeen uw gezondheid noemen?**

   (omcirkel één cijfer)

   Uitstekend..1
   Zeer goed..2
   Goed..3
   Matig..4
   Slecht...5

2. **In hoeverre hebben Uw lichamelijke gezondheid of emotionele problemen U gedurende de afgelopen 4 weken gehinderd in Uw normale omgang met familie, vrienden of buren, of bij activiteiten in groepsverband?**

   Helemaal niet..1
   Enigzins...2
   Nogal...3
   Veel..4
   Heel erg veel..5

3. Mentale Gezondheid en Vitaliteit

Deze vragen gaan over hoe u zich voelt en hoe het met u ging in de afgelopen vier weken.
Wilt u a.u.b. bij elke vraag het antwoord geven dat het best benadert hoe u zich voelde.

Scoremogelijkheden:

1	altijd
2	meestal
3	vaak
4	soms
5	zelden
6	nooit

Hoe vaak gedurende de afgelopen vier weken:

	Omcirkel één cijfer op elke regel					
Voelde u zich levenslustig?	1	2	3	4	5	6
Was u erg zenuwachtig?	1	2	3	4	5	6
Zat u zo in de put dat niets u kon opvrolijken	1	2	3	4	5	6
Voelde u zich rustig en tevreden?	1	2	3	4	5	6
Had u veel energie?	1	2	3	4	5	6
Voelde u zich somber en neerslachtig?	1	2	3	4	5	6
Voelde u zich uitgeput?	1	2	3	4	5	6
Was u een gelukkig mens?	1	2	3	4	5	6
Voelde u zich moe?	1	2	3	4	5	6

4. Hoe vaak hebben uw lichamelijke gezondheid of emotionele problemen U gedurende de afgelopen 4 weken gehinderd bij Uw sociale activiteiten (zoals vrienden of familie bezoeken, etc.)?

Omcirkel één cijfer op elke regel

Altijd..1
Meestal...2
Soms...3
Zelden..4
Nooit..5

5. Hoe JUIST of ONJUIST is elk van de volgende uitspraken voor U?

Omcirkel één cijfer op elke regel

	Volkomen juist	Grotendeels juist	Weet ik niet	Grotendeels onjuist	Volkomen onjuist
a. Ik lijk wat gemakkelijker ziek te worden	1	2	3	4	5
b. Ik ben even gezond als andere mensen	1	2	3	4	5
c. Ik verwacht dat mijn gezondheid achteruit zal gaan	1	2	3	4	5
d. Mijn gezondheid is uitstekend	1	2	3	4	5

3. INTRINISIC MOTIVATION INVENTORY (IMI)

Interesse/Genot

1. Ik heb erg genoten van deze arm-hand training

**1**	**2**	**3**	**4**	**5**	**6**	**7**
**helemaal niet waar**			**enigszins waar**			**zeer waar**

2. Deze arm-hand training was leuk

**1**	**2**	**3**	**4**	**5**	**6**	**7**
**helemaal niet waar**			**enigszins waar**			**zeer waar**

3. Ik vond deze arm-handtraining saai

**1**	**2**	**3**	**4**	**5**	**6**	**7**
**helemaal niet waar**			**enigszins waar**			**zeer waar**

4. Deze training kon mijn aandacht helemaal niet vasthouden

**1**	**2**	**3**	**4**	**5**	**6**	**7**
**helemaal niet waar**			**enigszins waar**			**zeer waar**

5. Ik vond deze arm-hand training interessant

**1**	**2**	**3**	**4**	**5**	**6**	**7**
**helemaal niet waar**			**enigszins waar**			**zeer waar**

6. Ik dacht dat deze arm-hand training vrij prettig was

**1**	**2**	**3**	**4**	**5**	**6**	**7**
**helemaal niet waar**			**enigszins waar**			**zeer waar**

7. Terwijl ik deze training deed, dacht ik na over hoe prettig ik het vond

**1**	**2**	**3**	**4**	**5**	**6**	**7**
**helemaal niet waar**			**enigszins waar**			**zeer waar**

Ervaren competentie

8. Ik denk dat ik vrij goed ben in deze training

1	2	3	4	5	6	7
**helemaal niet waar**			**enigszins waar**			**zeer waar**

9. Ik denk dat ik deze training vrij goed doe in vergelijking met andere deelnemers

1	2	3	4	5	6	7
**helemaal niet waar**			**enigszins waar**			**zeer waar**

10. Na een beetje ervaring met deze training, voelde ik me vrij bekwaam

1	2	3	4	5	6	7
**helemaal niet waar**			**enigszins waar**			**zeer waar**

11. Ik ben tevreden met mijn prestatie op deze trainingsactiviteit

1	2	3	4	5	6	7
**helemaal niet waar**			**enigszins waar**			**zeer waar**

12. Ik was vrij vaardig in het uitvoeren van deze trainingsactiviteit

1	2	3	4	5	6	7
**helemaal niet waar**			**enigszins waar**			**zeer waar**

13. Dit was een trainingsactiviteit die ik niet goed kon uitvoeren

1	2	3	4	5	6	7
**helemaal niet waar**			**enigszins waar**			**zeer waar**

Inspanning/Belangrijkheid

14. Ik heb veel moeite gedaan voor deze arm-hand training

1	2	3	4	5	6	7
**helemaal niet waar**			**enigszins waar**			**zeer waar**

15. Ik heb niet echt geprobeerd om goed te presteren op deze trainingsactiviteit

1	2	3	4	5	6	7
**helemaal niet waar**			**enigszins waar**			**zeer waar**

16. Ik heb me veel moeite gedaan tijdens deze trainingsactiviteit

1	2	3	4	5	6	7
**helemaal niet waar**			**enigszins waar**			**zeer waar**

17. Het was belangrijk voor mij om deze training goed te doen

1	2	3	4	5	6	7
**helemaal niet waar**			**enigszins waar**			**zeer waar**

18. Ik heb niet veel energie gestoken in de trainingsactiviteit

1	2	3	4	5	6	7
**helemaal niet waar**			**enigszins waar**			**zeer waar**

adelante
haal het beste uit jezelf

Druk/Spanning

19. Ik heb me helemaal niet zenuwachtig gevoeld terwijl ik de oefende

**1**	**2**	**3**	**4**	**5**	**6**	**7**
**helemaal niet waar**			**enigszins waar**			**zeer waar**

20. Ik heb me erg gespannen gevoeld terwijl ik oefende

**1**	**2**	**3**	**4**	**5**	**6**	**7**
**helemaal niet waar**			**enigszins waar**			**zeer waar**

21. Ik was zeer ontspannen tijdens het doen van deze oefeningen

**1**	**2**	**3**	**4**	**5**	**6**	**7**
**helemaal niet waar**			**enigszins waar**			**zeer waar**

22. Ik voelde me angstig tijdens het oefenen

**1**	**2**	**3**	**4**	**5**	**6**	**7**
**helemaal niet waar**			**enigszins waar**			**zeer waar**

23. Ik voelde dat ik onder druk stond tijdens het trainen

**1**	**2**	**3**	**4**	**5**	**6**	**7**
**helemaal niet waar**			**enigszins waar**			**zeer waar**

Waarde/Nut

24. Ik geloof dat deze activiteit waarde voor mij kan hebben

1	2	3	4	5	6	7
**helemaal niet waar**			**enigszins waar**			**zeer waar**

25. Ik denk dat deze activiteit nuttig is voor het verbeteren van arm-handvaardigheid

1	2	3	4	5	6	7
**helemaal niet waar**			**enigszins waar**			**zeer waar**

26. Ik denk dat deze training belangrijk is omdat ik mijn arm-hand meer en beter kan gebruiken

1	2	3	4	5	6	7
**helemaal niet waar**			**enigszins waar**			**zeer waar**

27. Ik zou dit nog wel eens willen doen omdat het enige waarde voor mij heeft

1	2	3	4	5	6	7
**helemaal niet waar**			**enigszins waar**			**zeer waar**

28. Ik denk dat het doen van deze activiteit mij zou kunnen helpen om mijn aangedane arm en hand meer te gebruiken in alledaagse activiteiten

1	2	3	4	5	6	7
**helemaal niet waar**			**enigszins waar**			**zeer waar**

29. Ik geloof dat het doen van deze training mij ten goede komt

1	2	3	4	5	6	7
**helemaal niet waar**			**enigszins waar**			**zeer waar**

30. Ik denk dat dit een belangrijke training is

1	2	3	4	5	6	7
**helemaal niet waar**			**enigszins waar**			**zeer waar**

EQ-HQ-APPEAL
Questionnaire on a 5-point scale

Begrijpelijk Niet begrijpelijk
| |. | | | | |

Ondersteunend Werkt tegen
| | | | | | |

Simpel Complex
| | | | | | |

Voorspelbaar Onvoorspelbaar
| | | | | | |

Duidelijk Verwarrend
| | | | | | |

Vertrouwd Niet betrouwbaar
| | | | | | |

Te beinvloeden Niet te beinvloeden
| | | | | | |

Bekend Vreemd
| | | | | | |

Interessant Saai
| | | | | | |

Duur Goedkoop
| | | | | | |

Spannend Vervelend
| | | | | | |

Exclusief Standaard
| | | | | | |

Indrukwekkend Nietszeggend
| | | | | | |

Origineel Gewoon
| | | | | | |

Innovatief Conservatief
| | | | | | |

Plezierig Niet plezierig
| | | | | | |

Goed Slecht
| | | | | | |

Esthetisch Niet esthetisch
| | | | | | |

Uitnodigend Afstotend
| | | | | | |

Aantrekkelijk Niet aantrekkelijk
| | | | | | |

Sympathiek Niet sympathiek
| | | | | | |

Motiverend Ontmoedigend
| | | | | | |

Gewenst Ongewenst
| | | | | | |
